# Exosomes Recovered From the Plasma of COVID-19 Patients Expose SARS-CoV-2 Spike-Derived Fragments and Contribute to the Adaptive Immune Response

**DOI:** 10.3389/fimmu.2021.785941

**Published:** 2022-01-17

**Authors:** Elisa Pesce, Nicola Manfrini, Chiara Cordiglieri, Spartaco Santi, Alessandra Bandera, Andrea Gobbini, Paola Gruarin, Andrea Favalli, Mauro Bombaci, Alessandro Cuomo, Federica Collino, Giulia Cricrì, Riccardo Ungaro, Andrea Lombardi, Davide Mangioni, Antonio Muscatello, Stefano Aliberti, Francesco Blasi, Andrea Gori, Sergio Abrignani, Raffaele De Francesco, Stefano Biffo, Renata Grifantini

**Affiliations:** ^1^ Istituto Nazionale Genetica Molecolare (INGM), Istituto Nazionale Genetica Molecolare “Romeo ed Enrica Invernizzi”, Milan, Italy; ^2^ Department of Biosciences, Università degli Studi di Milano, Milan, Italy; ^3^ Unit of Bologna, Consiglio Nazionale delle Ricerche (CNR) Institute of Molecular Genetics “Luigi Luca Cavalli-Sforza”, Bologna, Italy; ^4^ Istituto di Ricovero e Cura a Carattere Scientifico (IRCCS), Istituto Ortopedico Rizzoli, Bologna, Italy; ^5^ Infectious Diseases Unit, Fondazione Istituto di Ricovero e Cura a Carattere Scientifico (IRCCS) Ca’ Granda Ospedale Maggiore Policlinico, Milan, Italy; ^6^ Department of Pathophysiology and Transplantation, Università degli Studi di Milano, Milan, Italy; ^7^ Centre for Multidisciplinary Research in Health Science (MACH), Università degli Studi di Milano, Milan, Italy; ^8^ Department of Experimental Oncology, Istituto Europeo di Oncologia (IEO), European Institute of Oncology Istituto di Ricovero e Cura a Carattere Scientifico (IRCCS), Milan, Italy; ^9^ Laboratory of Translational Research in Paediatric Nephro-Urology, Fondazione Ca’ Granda IRCCS Ospedale Maggiore Policlinico, Milan, Italy; ^10^ Department of Clinical Sciences and Community Health, University of Milano, Milan, Italy; ^11^ Respiratory Unit and Cystic Fibrosis Adult Center, Respiratory Unit and Cystic Fibrosis Adult Center, Milan, Italy; ^12^ Department of Clinical Sciences and Community Health, Università degli Studi di Milano, Milan, Italy; ^13^ Department of Pharmacological and Biomolecular Sciences, Università degli Studi di Milano, Milan, Italy

**Keywords:** COVID-19, SARS-CoV-2, exosomes, immune activation, antigen-presenting cells (APCs), soluble mediators in immunity

## Abstract

Coronavirus disease 2019 (COVID-19) is an infectious disease caused by beta-coronavirus severe acute respiratory syndrome coronavirus 2 (SARS-CoV-2) that has rapidly spread across the globe starting from February 2020. It is well established that during viral infection, extracellular vesicles become delivery/presenting vectors of viral material. However, studies regarding extracellular vesicle function in COVID-19 pathology are still scanty. Here, we performed a comparative study on exosomes recovered from the plasma of either MILD or SEVERE COVID-19 patients. We show that although both types of vesicles efficiently display SARS-CoV-2 spike-derived peptides and carry immunomodulatory molecules, only those of MILD patients are capable of efficiently regulating antigen-specific CD4^+^ T-cell responses. Accordingly, by mass spectrometry, we show that the proteome of exosomes of MILD patients correlates with a proper functioning of the immune system, while that of SEVERE patients is associated with increased and chronic inflammation. Overall, we show that exosomes recovered from the plasma of COVID-19 patients possess SARS-CoV-2-derived protein material, have an active role in enhancing the immune response, and possess a cargo that reflects the pathological state of patients in the acute phase of the disease.

## Introduction

Coronavirus disease 2019 (COVID-19) primarily affects the lung epithelium, and infection may lead to pneumonia, respiratory distress syndromes, acute lung injury, and death. Severe acute respiratory syndrome coronavirus 2 (SARS-CoV-2) is a spherical or pleomorphic enveloped virus with a typical size range of 80–120 nm in diameter. It contains a positive single-stranded RNA of 30 kb, surrounded by a membrane embedded with several viral proteins. Of fundamental importance for viral internalization is the Spike (S) protein (1). During viral entry, the S protein binds, through its receptor-binding domain (RBD), to angiotensin-converting enzyme 2 (ACE2) receptors of host cells. Next, the serine protease TMPRSS2 primes the S protein for internalization (2). Once internalized, the virus matures, replicates, and, lastly, leaves the host cells in order to spread in the surrounding tissues. Many viruses are known to enter extracellular double-membrane vesicles (EDMVs) during intra-host spreading (3, 4). To date, however, literature covering this topic for SARS-CoV-2 is scanty.

Exosomes are small vesicles (30–100 nm in diameter) of endocytic origin and are released from cells into the extracellular environment during both normal and pathological conditions (5). They are formed by the inward budding of late endosomal membranes that give rise to intracellular multivesicular bodies (MVBs) that then fuse with the plasma membrane releasing the intraluminal exosomes into the extracellular space. They are secreted by all cell types and are present in bodily fluids, such as blood, urine, and saliva, breast milk, and bronchial and nasal lavage (6). Although the protein composition of exosomes reflects that of the parent cell, exosomes have common and peculiar components. They are in fact generally rich in tetraspanins (CD9, CD63, CD81) and heat shock and Rab proteins, which are routinely used as exosomal markers. Exosomes are an important tool for intercellular communication, as they act as shuttles for the transfer of biologically active proteins, lipids, and RNAs (7). *In vitro* studies have demonstrated that exosomes play a dual role: they promote pathogen transmission and exacerbate infection in some cases, while they contribute to host defense and control infection in others. Currently, very little is known about the characteristics, behavior, and contribution to viral infection of *in vivo* generated exosomes (8).

In this study, we purified exosomes from the plasma of COVID-19 patients using immuno-isolation methods and highlighted their function in the context of COVID-19 infection. In addition, we analyzed the differences in protein composition (internal cargo and membrane surface components) of exosomes recovered from healthy donors (HDs) and patients experiencing COVID-19 in a MILD or SEVERE form and elucidated possible functional roles.

## Materials and Methods

**Table:** 

Reagent or Resource	Source	Identifier
**Antibodies and Chemicals**		
Anti-SARS-CoV-2 Spike RBD	Sino Biological	40592-T62
4-μm-diameter latex beads	Invitrogen	A37304
Anti-human CD9 FITC	BD Biosciences	555371
Anti-human CD63 PE	BD Biosciences	556020
Anti-SARS-CoV-2 Spike S1 (Mab Clone #007)	Sino Biological	40150-R007
Anti-HSP70	Cell Signaling	4872
Anti-human CD19 APC-Cy7	BD Biosciences	557791
Anti-human CD68 FITC	BD Biosciences	562117
Anti-human CD86 PE-Cy7	BD Biosciences	561128
Anti-human HLA-DR APC-Cy7	BioLegend	307617
Anti-human HLA-DR PE-Cy5	BD Biosciences	555813
Anti-human CD11b FITC	BD Biosciences	562793
Anti-human CD11c PE-Cy5	BD Biosciences	551077
Anti-human CD25 PE	BD Biosciences	555432
Anti-human CD3 APC	BD Biosciences	555342
Anti-human CD56 APC	BD Biosciences	555518
Anti-human CD4 APC-Cy7	BD Biosciences	557871
Purified mouse anti-human HLA-DR, DP, DQ	BD Pharmingen™	555557
Anti-human TNFα FITC	BD Biosciences	552889
Anti-human IL-2 APC	BD Biosciences	555434
Anti-human IFNγ PerCP-Cy5.5	BD Biosciences	560704
EZ-Link Sulfo-NHS-LC-Biotinylation Kit	Thermo Scientific	21435
Streptavidin–horseradish peroxidase (HRP) conjugate	Invitrogen	SA10001
Dynabeads™ Protein G	Invitrogen	10009D
Alexa Fluor™ 647 Antibody Labeling Kit	Invitrogen	A20186
Abberior^®^ STAR RED	Abberior	STRED -1002
CD14 MicroBeads	Miltenyi Biotec	cat 130-050-201
CD4^+^ T Cell Isolation Kit	Miltenyi Biotec	cat 130-096-533
CellTrace™ Violet Cell Prolif. Kit	Invitrogen	C34557
Interleukin-2, human	Roche	10799068001
Anti-human CD4 BUV395	BD	564724
Anti-human HLA-DR BUV 650	BD	564724
qEV/35nm columns SEC Columns for Exosomes Separation and Purification	IZON	–
PreOmics “iST” Kit	PreOmics GmbH	P.O. 00001
High pH Reversed-Phase Peptide Fractionation Kit	Pierce™	84868
EasySpray PEPMAP RSLC C18	Thermo Fisher Scientific	ES801
Intracellular Fixation and Permeabilization Buffer Set	eBioscience	88-8824-00
Anti-human Syntenin (EPR8102)	abcam	ab236071
5-nm Gold-anti-Rabbit Fabs	BBI International	
Formvar/Carbon Film 10 nm/1 nm thick on Square 200 mesh Copper Grid	Electron Microscopy Sciences	FCF200CU50
Anti-human CD63 (Ts63)	Invitrogen	10628D
Anti-human CD9	Abcam	92726
Anti-human TSG101 (4A10)	Abcam	Ab83
Anti-ApoA (EPR2949)	Santa Cruz	sc-376818
**Critical commercial assays**		
ExoTEST Ready to Use Kit for Overall Exosome capture and quantification from human plasma	Hansa BioMed	–
ExoView™ Tetraspanin chip	Nanoview Biosciences	
**Software**		
FACSDiva	BD Biosciences	
FlowJO		v.10.6.2
ExoViewer		
MaxQuant (MQ)		v.1.6.10.43
NTA 3.4 software	Malvern Instruments	v.3.4
Perseus		
Heatmapper		
FunRich		v.3.1.4
R Studio	GNU	v4.0.3
topGO49	R package	v2.42.0
ggplot	R package	v3.3.3
Cytoscape		v3.8.2
STRINGapp		
Omics visualizer app		
ImageJ	NIH	
iTEM		

FITC, Fluorescein isothiocyanate; PE, Phycoerythrin; APC-Cy7, Allophycocyanin-Cy7; PE-Cy7, Phycoerythrin-Cy7; PE-Cy5, Phycoerythrin-Cy5; APC, Allophycocyanin; PerCP-Cy5.5, Peridinin chlorophyll)-Cy5.5; BUV395, Brilliant Ultraviolet 395; BUV650, Brilliant Ultraviolet 650; Hsp70, Heat shock protein 70; HLA-DR, Human Leukocyte Antigen-DR -DP -DQ isotype; TNFα, Tumor necrosis factor alpha; IL-2, Interleukin-2; IFNγ, Interferon gamma; SEC, Size Exclusion Chromatography; TSG101, tumour susceptibility gene 101; ApoA, Apolipoprotein A.

### Sample Collection

Plasma samples from COVID-19 patients were collected by the Ospedale Maggiore Policlinico (Milan, Italy) between March and June 2020 in the acute phase of infection from venous blood samples using Ethylenediaminetetraacetic acid (EDTA) as anticoagulant. The clinical classification of MILD and SEVERE patients was done following the recommended WHO clinical progression scale score (MILD patient scores ranged between 1 and 4, whereas SEVERE patients had scores between 5 and 10) (9). Detailed information is shown in **Supplementary Table S1**. For the SEVERE group, samples were collected during patients’ hospitalization in COVID-19 high dependency units. COVID-19 patients were diagnosed by RT-PCR nasopharyngeal swab. HDs were chosen to be representative of both sexes and of varied ages (26–65 years). While gender was matched between COVID-19 patients and healthy controls in this cohort, the average age of the SEVERE and MILD group was not, the first being much higher than the second, following an intrinsic characteristic of COVID-19 epidemology that shows a worse prognosis in elderly individuals.

### Human Plasma Preparation

In this study, 100 µl of plasma samples were prepared by 3 centrifugation steps (Step 1: 10′ at 500 g; Step 2: 20′ at 2,000 g; Step 3: 30′ at 14,000 g) to eliminate red blood cells and cellular debris. After each step, the supernatant was transferred to a new tube and the pellet was discarded. After the last centrifugation, the supernatant was diluted in Phosphate-buffered saline (PBS). To exclude cell-free virions in the exosome preparations, we always used immune affinity purification method using anti-tetraspanin antibody-conjugated magnetic or latex beads.

### Immunocapture-Based ELISA

Circulating exosomes from plasma of MILD and SEVERE patients or HDs were captured using the ExoTEST Kit (Hansa BioMed) according to the manufacturer’s instructions. The ELISA plate with 100-µl test samples loaded per well was incubated at room temperature with shaking for 30 min. After washing 3 times with washing buffer, 100 μl of biotinylated anti-SARS-CoV-2-S RBD primary antibody (1:1,000 ratio) were added to each well and incubated at room temperature while shaking for 2 h (2–3 rotations per second). The plate was washed again with the washing buffer, and 100 μl of diluted streptavidin HRP-conjugated were added to each well. The plate was incubated at room temperature while shaking for 1 h (2–3 rotations per second). Then, 100 μl of Substrate Chromogenic Solution was added to each well and incubated uncovered at room temperature in the dark for 5–10 min. The plate was monitored until a blue color was visible. At this point, the reaction was stopped by adding 100 μl of stop solution to each well. The absorbance was recorded at 450 nm within 10 min with an Infinite F200 (Thermo Fisher Scientific, USA).

### Flow Cytometry Analysis

For immunoisolation of exosomes recovered from plasma, 10 μl of 4-μm-diameter latex beads were incubated with 20 µl purified anti-CD63 mAb for 30 min at room temperature in a final volume of 50 μl. After 15 min, the volume was made up to 200 μl with PBS, and samples were incubated overnight at 4°C with gentle agitation. For Fluorescence activated cell sorting (FACS) analysis, exosomes recovered from blood plasma were incubated in 60 μl for 30 min at 4°C with anti-CD63-latex beads. The volume was made up to 400 μl with PBS and incubated for 2 h at 4°C. To eliminate the unspecific antibody binding, beads resulting after plasma incubation were blocked with 5% bovine serum albumin (BSA) solution for 30 min at room temperature. Vesicle-coated beads were washed twice in washing buffer (1% BSA in PBS) and resuspended in 400 μl of washing buffer, stained with the indicated fluorescent antibodies, and analyzed on a FACSCantoI flow cytometer (BD Biosciences) and FACSDiva software. Flow cytometry analysis was done interchangeably using an isotype antibody or a secondary antibody control.

For exosome integrity analysis, we also assessed syntenin compartmentalization in the exosome cargo. The Intracellular Fixation and Permeabilization Buffer Set (eBioscience) was used to fix and permeabilize exosomes for intravesicular staining. Exosomes were fixed in 100 μl of IC Fixation Buffer and incubated in the dark at room temperature for 20 min. After incubation, the samples were washed twice with 500 μl of permeabilization buffer and then resuspended in 100 μl of the same buffer. The samples were then incubated with PE anti-human syntenin for 30 min at room temperature. After incubation, the samples were washed twice with 500 μl of permeabilization buffer and resuspended in 200 μl of 1% BSA-PBS and analyzed.

### Nanoparticle Tracking Analysis

The size distribution and concentration of plasma-derived exosomes were analyzed by nanoparticle tracking analysis (NTA) using NanoSight model NS300 equipped with a Blue488 laser and a sCMOS camera (Malvern Instruments, Malvern, UK). Here, 2 μl of sample was diluted in 1 ml of PBS and then injected into the laser chamber. The following settings were used for data acquisition: camera level, 13; acquisition time, 60 s; and detection threshold, 5. Data were analyzed using the NTA 3.4 software (Malvern Instruments). Three recordings were performed for each sample. The evaluation of the particle size distribution (PSD) was performed through the following parameters: Mean, Mode, SD, D10, D50 (Median), and D90, which indicate, respectively, the average, most frequent particle class size, standard deviation, and the 10%, 50%, and 90% percentiles of the analyzed particles.

### Exosome Coimmunoprecipitation Followed by Western Blotting

In this study, 5 μg of anti-CD63, anti-CD9, and anti-CD81 mAbs, diluted in 200 μl PBS, were added to 1.5 mg (50 μl) of the protein G-coated Dynabeads^®^ suspension and incubated with rotation for 20 min at room temperature. The Abs-coated beads were separated from the non-bound antibodies by placing the tube on a magnet for 1 min and removing the supernatant. Beads–Ab complexes were resuspended in 200 μl PBS with Tween^®^-20 and washed by gentle pipetting. Plasma samples (100 μl) were added to the Dynabeads^®^–Ab complexes, and the beads were incubated with rotation Overnight (O/N) at 4°C. The Dynabeads^®^–Ab–antigen complexes were washed 3 times using 200 μl PBS and were resuspended in 100 μl PBS. The bead suspension was then transferred to a clean tube to avoid co-elution of proteins bound to the tube wall. The tube was then placed on a magnet for 1 min, and the beads were recovered. Next, 50 μl of premixed NuPAGE^®^ LDS Sample Buffer and NuPAGE Sample Reducing Agent (mixed as per manufacturer’s instructions) were added to the Dynabeads^®^–Ab–antigen complex and heated for 10 min at 70°C. Then, the tube was placed on a magnet for 1 min, and the supernatant/sample was loaded onto a NuPAGE 4%–12% Bis–Tris gel (Invitrogen, Waltham, MA, USA) and transferred onto a Polyvinylidene fluoride (PVDF) membrane (Merck Millipore, Burlington, MA, USA). Membranes were blocked in 5% non-fat powdered milk in TBS-T (0.5% Tween-20) and probed with the indicated antibodies for 2 h at room temperature. Membranes were washed, and HRP-conjugated antibodies were added for 1 h at room temperature. Detection was performed using enhanced chemiluminescence (ECL) reagents (Invitrogen) according to the manufacturer’s guidelines.

### Exoview Analysis

Exosomes recovered from plasma of COVID-19 patients and HDs were analyzed using ExoView Tetraspanin chips (NanoView Biosciences, Boston, MA, USA) arrayed with antibodies against the CD81, CD63, CD9, and CD41a proteins. Mouse IgG1 was used as a negative control. In brief, 35 µl of each sample were dropped onto the chip surface (placed in a sealed 24-well plate) and incubated for 16 h at room temperature. Each chip was then washed once on an orbital shaker with PBST (PBS supplemented with 0.05% Tween-20) for 3 min, then washed three additional times with PBS for 3 min. After washing, the chips were incubated with anti-SARS-CoV-2-S RBD, conjugated with Alexa Fluor 647, in PBST supplemented with 2% BSA in a volume of 250 μl for 2 h at room temperature without shaking. Next, each chip was washed once with PBST, 3 times with PBS, once in filtered deionized water, and then dried at room temperature for 1 h. The chips were then imaged with the ExoView R100 reader (ExoView) and analyzed using the ExoViewer software with a sizing threshold set to 50–200-nm diameter. The resulting size and fluorescence intensity information for each individual exosome was exported to Excel for statistical analyses. Fluorescence values are reported in arbitrary units.

### Stimulated Emission Depletion Microscopy

For SARS-CoV-2-S immunolabeling and stimulated emission depletion (STED) microscopy evaluation, latex bead-isolated exosomes were used, similarly to what was previously described for FACS immunostaining. Exosomes prepared from blood plasma of HD and MILD and SEVERE COVID patients were incubated with anti-CD63-latex beads, and beads were then blocked with 5% BSA solution to eliminate unspecific antibody binding. Vesicle-coated beads were washed and seeded onto glass coverslips (n. 1.5 thickness; electron microscopy), then stained with anti-SARS-CoV-2-S RBD (1:500), followed by hybridization with secondary antibodies conjugated with the Star*RED fluorophore (Abberior; 1:200), and finally mounted onto glass slides with ProlongGlass mounting reagent for super-resolution (Molecular Probes, Thermo Fisher Scientific). Samples were acquired using a demo version of the Abberior STEDYCON microscope for simultaneous confocal and STED microscopy (kind collaboration with Abberior Instrument) with 4 excitation laser lines and a 775-nm depletion STED laser. Star*RED fluorophore excitation was kept at 10% power of the 640-nm laser power, whereas Star*RED fluorophore depletion was obtained at 100% laser power in order to achieve 30-nm resolution, at pixel size 15 nm, with 7 lines of STED acquisition over different Z-plan, spanning throughout the whole z-dimension of the latex beads. A total of n = 9 beads were acquired in STED imaging for all conditions (HD, MILD, SEVERE) with duplicate independent biological replicates, reaching a total of n = 18 for each condition. STED-resolved microscopy images were quantified upon precise photon counting. Data were normalized with subtraction of photon counts of background signal, as evaluated by anti-SARS-CoV-2-S RBD–Star*RED in HD samples, with mean photon count in raw images =44, whereas mean photon count in raw images from MILD and SEVERE samples were =152 and =80, respectively.

### Transmission Electron Microscopy

Transmission electron microscopy evaluation of RBD immunogold labeling was performed on HD, MILD COVID-19, and SEVERE COVID-19 plasma-derived exosomes purified *via* lattice-bead immunocapture as described in the previous sections and stained in suspension with rabbit anti-SARS-CoV-2-S RBD (1:500) followed by 5-nm Gold-anti-Rabbit Fabs (BBI International) (1:200). After staining, samples were fixed in 1% glutaraldehyde and finally kept in PBS pending TEM observation. TEM sample preparation was performed similarly to what has been recently described (10). Here, 5 μl of the pooled EV sample was layered onto a formvar/carbon-coated 200 mesh grids and allowed to settle for 20 min. No further negative stain was performed in order to preserve a proper contrast for immunogold labeling. The grids were blotted and allowed to air-dry at room temperature. The observations were carried out with a JEOLJEM-1011 (Jeol Jem, Peabody, MA, USA) transmission electron microscope operated at 100 kV. Images were captured using a Morada G2 TEM digital camera (Olympus Soft Imaging Solutions, Münster, Germany) and iTEM software. Negative controls (not shown) were performed in the absence of primary antibodies. Here, 10–15 images were captured from each of three randomly selected areas of each grid at ×50,000 and ×100,000 lens magnification. The camera magnifications were calibrated using a grid with a grating replica (EMS cata #80050) with line spacing of 463 nm (2,160 lines/μm). Scale bars reflect the magnification at the camera. TEM micrographs were analyzed manually. Rounded or “cup-shaped” particles with high-contrast edges were considered exosomes and measured using Fiji-ImageJ (with Java 1.8.0_172, 64-bit).

### STOchastic Reconstruction Microscopy

Single-molecule super-resolution microscopy for RBD fluorescent immunolabeling on MILD COVID plasma-derived exosomes was performed with direct stochastic optical reconstruction microscopy (D-STORM) modality (i.e., spontaneous particle blinking with only excitation in fluorophore reporter wavelength). Exosomes were purified *via* lattice-bead immunocapture as previously described and stained in suspension with rabbit anti-SARS-CoV-2-S RBD (1:500) followed by AlexaFluor-647 Goat anti-Rabbit IgGs (Molecular Probes, Thermo Fisher) at 1:200. After staining, samples were fixed in 1% Paraformaldehyde (PFA) for fluorophore preservation and finally kept in PBS pending STORM assay. For acquisition, 5 μl of the labeled exosome pool were carefully layered at the center of a glass-inserted 35-mm petri dish (CellView) and air-dried under the laminar hood for 30 min to achieve a correct glass-surface deposition of labeled exosomes before adding 250 μl of freshly prepared STORM buffer (Abbelight) and topping the sample with a 22-mm square glass. The achieved volume of buffer of acquisition surface was optimized for correct evanescence wave formation within total internal reflection fluorescent (TIRF) illumination. Acquisitions were performed on an N-STORM instrument (Nikon Instruments) mounted on a Nikon Ti widefield with DU-897 EM-CCD camera (Andor Technology) with TIRF illuminator and ×100 TIRF (NA 1.49) objective (Nikon Instrument), coupled with 10-mW 647 excitation/reported laser (CrystaLaser) used at 70% power for D-STORM for 10,000 frames/acquisition over a constant TIRF-plane angle, with 1 frame exposure detection (at 10–20 ms range) at 17 Mhz, for both 2D and 3D STORM. After acquisitions, particle-detection data reconstruction was obtained with the *ad hoc* STORM analysis module in NIS-Elements v.5.31 (Lim-Nikon Instruments) with the following parameters for blinking molecule detection over acquired frames: standard Gaussian fitting for the localization of specific molecules characterized by minimum bit Height 500, maximum bit Height 65535, over a CCD bit baseline 100, for specific counting of molecules with minimum width 20 nm, maximum width 200 nm, initial fit width 100 nm. For 2D STORM, the x and y coordinates from each blinking molecule were retrieved for molecule localization; for 3D STORM, the 3 coordinates (x, y, z) were retrieved and 3D volume viewing was performed.

### CD4^+^ T-Cell Activation

CD14 monocytes purified from peripheral blood mononuclear cells (PBMCs) by positive immunomagnetic sorting were loaded with 10 × 10^7^ of purified MILD patient-recovered exosomes for 6 h with rotation at 37°C. After incubation, monocytes were washed with RPMI containing 10% serum (exosome free), irradiated, cocultured with autologous negative immunomagnetic purified CD4^+^ T cells, and labeled with cell trace. Interleukin (IL)-2 was added after 48 h of culture. After 4 days, cells were stained with anti-CD4/BUV395 and anti-HLA-DR/BUV 650 and analyzed by flow cytometry (BD Symphony).

### Exosome Purification for Mass Spectrometry

Exosomes were purified from the plasma of 3 HDs, 4 patients presenting MILD COVID-19 symptoms, and 4 patients presenting SEVERE COVID-19 symptoms using qEV SMART Size Exclusion Chromatography (SEC) columns following manufacturer’s instructions. Briefly, 150 ul of plasma were centrifuged at 1,500 g for 10 min in order to remove cells and large contaminant particles. After a subsequent centrifugation at 10,000 g for 10 min, supernatants were loaded on IZON qEV/35nm columns, previously washed and equilibrated with 3 volumes of PBS. After column loading, samples were eluted with PBS using a void volume of 1 ml and an elution volume of 600 µl. Purified exosomes were then concentrated and processed for mass spectrometry analysis.

### Mass Spectrometry of Purified Exosomes

Quantitative proteome profiling from exosomes was achieved by a library-based approach combined with MS boxcar acquisition method in a label-free experiment as reported before (11). We constructed three different libraries by pooling proportional quantities of different exosome preparations from each sample type (MILD, SEVERE, and HD) assessed by Bicinchoninic acid (BCA) protein quantitation assay. Samples were run independently and then aligned to their respective libraries. In brief, proteins were denatured, reduced, alkylated, and digested, and peptides were purified on StageTips (12) using reagents from the PreOmics “iST” Kit (P.O. 00001, PreOmics GmbH). Only samples for the library were fractionated using a commercial High pH Reversed-Phase Peptide Fractionation Kit (Thermo Scientific). In all cases, dried peptides were reconstituted in 5 µl of LC-LOAD buffer (P.O. 00001, PreOmics GmbH). Samples were measured using liquid chromatography–mass spectrometry (LC-MS) instrumentation consisting of an EASY-nLC 1200 system coupled to a nano-electrospray ion source and a Q Exactive HF Orbitrap (all Thermo Fisher Scientific). Purified peptides were separated on an EasySpray PEPMAP RSLC C18 (Thermo Fisher Scientific) kept at 45°C constant to reduce column back pressure. Solvent A was 0.1% formic acid (FA), and solvent B was 0.1% FA in 80% Acetonitrile (ACN). Samples were loaded in aqueous 0.1% (FA) solution at constant pressure of 980 Bar. Peptides were separated with a gradient of 3%–30% solvent B over 39 min followed by a gradient of 30%–60% for 5 min and 60%–95% over 1 min at a flow rate of 300 nl/min. For the library, standard DDA experiments were performed with a data-dependent top15 method. In brief, MS spectra (from m/z 375–1,550) were analyzed in the Orbitrap detector with resolution R = 60,000 at m/z 200. The 10 most intense peptide ions with charge states ≥2 were sequentially isolated to a target value of 3e6 and fragmented by Higher Energy Collision Dissociation (HCD) with a normalized collision energy setting of 28%. The maximum allowed ion accumulation times were 20 ms for full scans and 80 ms for MSMS, and the target value for MSMS was set to 1e5. The dynamic exclusion time was set to 20 s. Unfractionated peptides corresponding to the study samples were injected in single-shot analysis (number of replicates equal to 4) with a BoxCar scan method where instrument acquisition was controlled by MaxQuant Live software (version 1.2) keeping default scan protocol parameters (13).

### Quantification and Statistical Analysis

Acquired raw data obtained by mass spectrometry analysis were analyzed using the MaxQuant (MQ) (14) version 1.6.10.43, and peptide lists were searched against the human Uniprot FASTA database (74470 Entries) with the Andromeda search engine (15). The main search was performed with an initial mass tolerance of 7 ppm. False discovery rate (FDR) for both protein and peptide identifications was set to a maximum of 1% with enzyme specificity set to Trypsin/P. A maximum of 2 missed cleavages was allowed, and the minimum peptide length was fixed at 7 amino acids and Carbamidomethylation of Cysteine was specified as a fixed modification. Peptides were identified with an initial precursor mass deviation of 7 ppm and a fragment mass deviation of 20 ppm. “Match between run algorithm” (MBR) in MaxQuant (16) was performed after constructing a matching library consisting of polled samples. For label-free protein quantitation (LFQ), we required a minimum ratio count of 2 (17). All proteins and peptides matching to the reversed database were filtered out. ProteinGroups.txt table from MQ output was analyzed using Perseus platform version (18). In brief, two-sample Student’s t-test was used to determine the significantly changed proteins between disease and control groups with a permutation-based FDR of 5%. For significant hits, minimal fold changes together with p values (controlled by the s0 parameter in Perseus) were used with a permutation-based FDR of 0.05 resulting from an s0 set to 0.1. Heatmap was generated using Heatmapper (19). Proteins were hierarchically clustered by setting Euclidean distance and average linkage method as parameters. Specific biological process enrichment and their plotting were generated in R Studio using R v4.0.3 and topGO (20) v2.42.0 passing the weight algorithm and Fisher test to the runTest function. A cutoff of 0.05 was applied, and the top 18 enriched terms were visualized with ggplot v3.3.3 (21). Protein–protein interaction (PPI) networks of unique upregulated and downregulated proteins are retrieved using the online version of Search Tool for the Retrieval of Interacting Genes/Proteins (STRING) database version 11.0, setting the maximum number of interactions for the first and second shell to not more than 10 interactors. Generated networks are imported in Cytoscape v3.8.2, where functional enrichment was performed using STRINGapp enrichment. Gene Ontology (GO) term sorting is performed by combining strength and number of input proteins retrieved for the term. Omics visualizer app (22) is used to show biologically relevant terms. For the other comparative analyses, two-tailed t-test and ANOVA test were used.

## Results

### Exosomes Recovered From the Plasma of MILD COVID-19 Patients Carry a Higher Amount of SARS-CoV-2-S-Derived Peptides Compared to Those of SEVERE COVID-19 Patients

**Figure 1:**
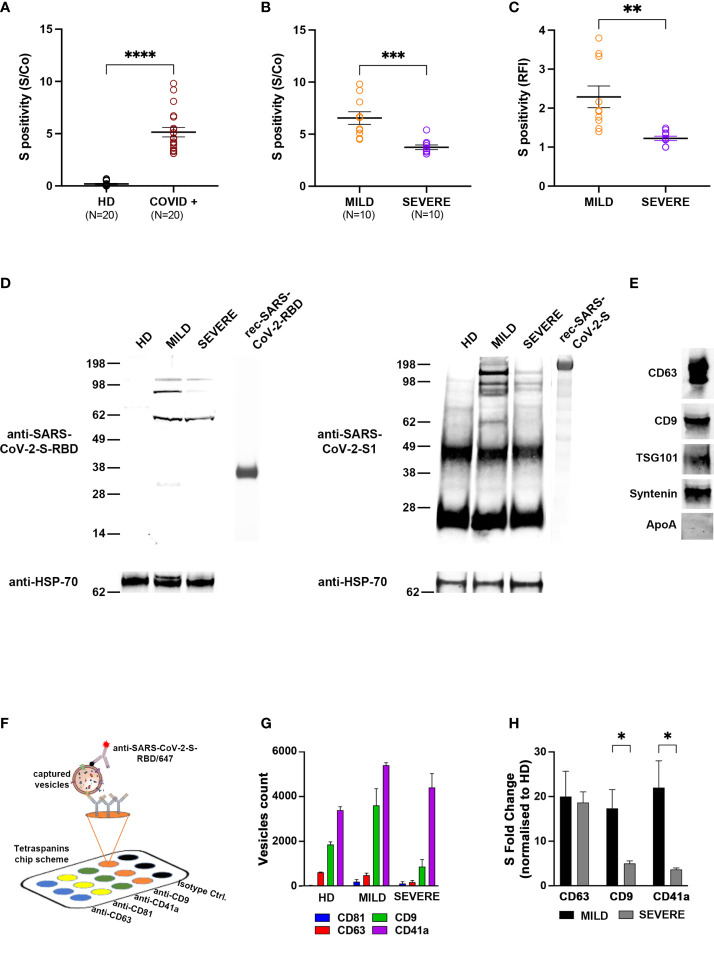
Circulating exosomes of MILD COVID-19 patients carry more SARS-CoV-2-S-derived peptides than those of SEVERE COVID-19 patients and are enriched in CD9 and CD41a exosomal markers. **(A, B)** Tetraspanin ELISA assay performed on 20 COVID-19 patients (10 with MILD symptoms and 10 with SEVERE symptoms) and 20 COVID-19-negative HDs. Patient-recovered samples were first analyzed as a whole **(A)** and subsequently separated based on class **(B)**. Positivity for S protein was calculated as the presence of the signal compared to a control well (S/Co) (Two-tailed t-test ****p < 0.0001). **(C)** Flow cytometry analysis. Latex beads coated with the anti-CD63 antibodies were incubated with plasma-recovered exosomes. Bead-bound exosomes were subjected to flow cytometry. Anti-CD9/AF488 antibodies were used to define and gate the specific exosome population. The percentage of exosomes positive for anti-SARS-CoV-2-S-RBD/APC is reported in RFI (value vs. isotype control) (One-way ANOVA with Tukey test ****p < 0.0001). **(D)** Western blot showing anti-SARS-CoV-2-S-RBD (left) and anti-SARS-CoV-2-S1 immunoblotting (right) in exosomes of COVID-19 patients or HDs. Exosomes were immunoprecipitated with anti-tetraspanin (CD63, CD9, CD81) antibodies and immunoblotted for the indicated proteins. Exosomal marker HSP70 was used as a loading control. Blots are representative of three independent experiments. **(E)** Western blots showing the presence of exosomal markers CD63, CD9, TSG101, and syntenin and absence of contaminant plasma-protein ApoA. **(F)** Scheme of the ExoView™ tetraspanin chip. EVs from plasma of COVID-19 patients or HDs were immobilized on ExoView™ chips by affinity capture against CD81, CD63, CD9, and CD41a exosomal transmembrane proteins. Once affinity-captured, the samples were incubated with fluorescent anti-SARS-CoV-2-S-RBD antibodies and analyzed using ExoView™ R100. **(G)** Difference in the average particle count from each antibody referred to in panel **(A)**. **(H)** Colocalization of SARS-CoV-2-S on the surface of the vesicles captured on the chip with the indicated antibodies. Histograms represent the count number (expressed as protein fold change) of SARS-CoV-2-S^+^ exosomes (t-test *p < 0.05, **p < 0.01, ***p < 0.001). HD, Healthy donor; S/Co, Signal/Control; RFI, Relative Fluorescence Intensity; S, Spike; rec, recombinant; Hsp70, Heat shock protein 70; TSG101, tumour susceptibility gene 101; ApoA, Apolipoprotein A; AF488, Alexa Fluor 488; APC, Allophycocyanin; Ctrl, Control.

In order to characterize exosomes of different COVID-19 patients, plasma from 20 individuals who tested positive for SARS-CoV-2 infection by nasopharyngeal swab real-time PCR was collected in the acute phase of the disease (within 21 days from the diagnosis). Overall, we enrolled 11 males and 9 females with a median age of 57.5 years [interquartile range (IQR) 27.5–70] and a median time from diagnosis of 14 days (IQR 14–16). Patients with a maximal WHO clinical progression score [https://doi.org/10.1016/S1473–3099(20)30483–7] between 1 and 4 were classified as MILD, whereas those with a maximal score between 5 and 10 were classified as SEVERE (9) (**Supplementary Table S1**). The selected MILD patients never transited to a SEVERE state during the course of the disease. As a control group, we enrolled 20 COVID-negative HDs. At first, we tested whether exosomes recovered from plasma of COVID-19 patients exposed any SARS-CoV-2-derived peptide. By using ExoTEST™, a platform for exosome quantification and characterization (23–25), and commercial anti-SARS-CoV-2-S antibodies for the detection of the S protein, we found that the SARS-CoV-2-S protein or derived fragments were clearly present in exosomes of COVID-19 patients but not in those of HDs, as expected (**Figure 1A**). Next, we subgrouped COVID-19 patient exosomes into MILD and SEVERE and analyzed whether SARS-CoV-2-S was more abundant in one of the two classes. Intriguingly, MILD patients had a higher amount of circulating SARS-CoV-2-S^+^ exosomes compared to patients with SEVERE symptoms (**Figure 1B**). To confirm this unexpected result, we recovered extracellular vesicles (EVs) from the plasma of either COVID-19 patients or HDs through sequential centrifugation and, after validating their size through NTA (**Figure S1A**), we used two alternative exosome isolation/characterization approaches: one based on anti-CD63-conjugated bead purification, followed by flow cytometry using anti-SARS-CoV-2-S-RBD antibodies for S protein detection (**Figure S1B**) and the other based on immunoprecipitation using anti-tetraspanins (CD63, CD9, CD81), followed by Western blotting using both anti-SARS-CoV-2-S RBD and anti-SARS-CoV-2-S1 antibodies (the latter specific for S protein subunit 1) in order to confirm the origin of the fragments detected. Flow cytometry confirmed that anti-SARS-CoV-2-S RBD antibodies bound exosomes of MILD COVID-19 patients with higher affinity compared to those of SEVERE patients, while they showed negligible binding to HD exosomes (**Figure 1C** and **Figure S1C**). Integrity of the analyzed exosomes was assessed by probing for cytosolic exosomal marker syntenin before and after permeabilization. Syntenin was detectable only after permeabilization, as expected for fully intact exosomes (**Figure S1D**). Western blotting revealed that both anti-SARS-COV-2-S RBD and anti-SARS-CoV-2-S1 antibodies recognized protein bands predominantly in exosomes of MILD patients. These bands had molecular weights (MWs) lower than 130 kDa (**Figure 1D**), thus corresponding to degradation fragments of the S protein, whose full-length monomer is 180 kDa. Purity of exosomal preparations was confirmed by positive detection of canonical exosomal markers, namely, CD63, CD9, TSG101, and syntenin, and lack of contaminant plasma proteins (i.e., ApoA) (**Figure 1E**).

Taken together, these results suggest that SARS-CoV-2-S-derived fragments are present in exosomes of all COVID-19 patients but are specifically and preferentially enriched in those of patients classified as MILD.

### SARS-CoV-2-S^+^ Exosomes Are Enriched in CD9 and CD41a

Next, we addressed whether the presence of SARS-CoV-2-S-derived peptides was associated to specific exosomal subpopulations by using the ExoView™ platform, which allows for single particle interferometric imaging measurements and analysis of differential coexpression of exosomal markers (26, 27). Exosomes were captured using antibodies against the canonical CD9, CD63, and CD81 exosome markers and platelet-specific CD41a (**Figure 1F**). CD41a was added to our strategy, since platelets function as an important exosomal source and are known to be hyperactivated in COVID-19 patients (28). By label-free detection, we observed that most of the exosomes were captured by anti-CD9 and anti-CD41a antibodies, and intriguingly, exosomes of MILD COVID-19 patients were remarkably more abundant compared to those of both SEVERE patients and HDs (**Figure 1G**). In addition, we found that exosomes from COVID-19 patients had an increased size distribution compared to HD exosomes, independently of the specific marker analyzed (**Figure S2**). Interestingly, CD41a^+^ exosomes of MILD COVID-19 patients were larger (50–160 nm) than those of SEVERE COVID-19 patients (50–100 nm) (**Figure S2**, top), possibly indicating a different cargo for the two exosome groups. Next, we wanted to assess whether the SARS-CoV-2-S-derived fragments were evenly distributed in exosomes or enriched in one of the four exosome subpopulations. We found that SARS-CoV-2-S was detected in all the exosomes analyzed, except for the CD81^+^ subpopulation (**Figure 1H**). When comparing MILD and SEVERE COVID-19 patient-recovered exosomes, we found that SARS-CoV-2-S peptides were drastically more enriched in the CD41a^+^ and CD9^+^ subpopulations of those recovered from MILD patients (**Figure 1H**).

**Figure 2:**
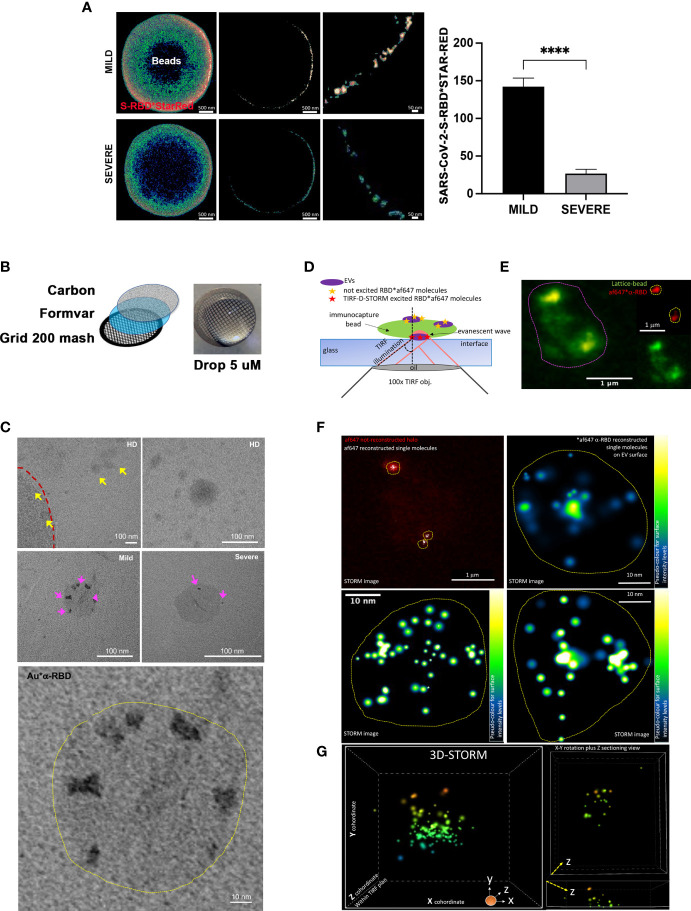
Imaging analysis confirms the increased presence of SARS-CoV-2-S on the surface of exosomes recovered from MILD COVID-19 patients **(A)** STED analysis of SARS-CoV-2-S expression in bead-captured exosomes. (Left) Representative image of one exosome-capturing latex bead from MILD (top) and SEVERE (bottom) COVID-19 patient samples acquired *via* STED microscopy using anti-SARS-CoV-2-S-RBD labeling. Images represent: (left) a single focal plan toward the top of the bead, (center) a perfect orthogonal Z-plan of the bead displaying its transactional external rim, (right) a further zoomed-in detail of the bead. (Right). Quantification of SARS-CoV-2-S-RBD*STAR-RED photon counts acquired *via* STED derived from a total of n = 149 beads (n = 45, n = 53, n = 51 beads from HD, MILD, and SEVERE samples, respectively) from n = 2 independent biological experiments. The bar chart represents means ± SEM for each sample per condition (MILD samples = 142 ± 22 photons and SEVERE samples = 27 ± 11 photons). Values are normalized with subtraction of photon counts from the background signal (evaluated by anti-SARS-CoV-2-S-RBD*STAR-RED in HD samples) (Paired t-test, ****p < 0.0001). **(B)** Scheme of TEM sample preparation *via* suspension-stained sample dripping onto carbon-coated Formavar grids. No embedding nor cutting was performed. **(C)** Representative TEM micrographs at lower and higher magnification of anti-SARS-CoV-2-S-RBD*gold labeled exosomes recovered from plasma of HD (upper row) or MILD (middle row left and lower enlarged image) or SEVERE (middle row right) COVID-19 patients immunocaptured with lattice beads. The external rim of the lattice 4-µm diameter bead is visible in the upper left image in panel **(C)** and highlighted by the red dotted line. Yellow arrowheads point to several HD exosomes negative for anti-SARS-CoV-2-S-RBD*gold staining. Magenta arrowheads point to positive anti-SARS-CoV-2-S-RBD*gold labeling, better detailed in the lower highly magnified image. **(D)** Scheme of single-molecule localization microscopy *via* TRF/direct-STORM on conjugated immunolabeled RBD on the surface of exosomes recovered from MILD COVID-19 patient plasma, immunocaptured *via* lattice beads. **(E)** Diffracted-limited widefield image of one representative autofluorescent bead (contoured by magenta dotted circle) capturing several af647-RBD-labeled exosomes. **(F)** STORM acquisition and reconstruction of blinking af647-anti-RBD molecules from the chosen bead shown in panel **(D)** Yellow dot circles indicate the areas occupied by RBD^+^ exosomes. Single molecules are shown either in white or in pseudo-coloring enlarged images of n = 3 chosen RBD^+^ exosomes. **(G)** 3D-STORM acquisition and reconstruction with x, y, z coordinates of single molecule localization within the TIRF plan for 3D view of a representative MILD COVID-19 plasma-recovered exosome immunolabeled for RBD. Single molecules are shown in 16-ramp pseudo-color scale. HD, Healthy donor; S/Co, Signal/Control; RFI, Relative Fluorescence Intensity; S, Spike; rec, recombinant; Hsp70, Heat shock protein 70; TSG101, tumour susceptibility gene 101; ApoA, Apolipoprotein A; AF488, Alexa Fluor 488; APC, Allophycocyanin; Ctrl, Control.

This result was confirmed using STED microscopy, a fast-performing super-resolution technique for resolving objects smaller than the light diffraction limit. By visualizing and quantifying the levels of SARS-CoV-2-S fragments on bead-purified CD9^+^ exosomes, we confirmed a significant enrichment of SARS-COV-2-S peptides in MILD COVID-19 patient compared to SEVERE COVID-19 patient exosomes (**Figure 2A** and **Figure S3**).

Next, to further validate this result at nanometer levels, we performed TEM using a suspension immunogold labeling protocol optimized to detect SARS-CoV-2-S peptides on immunocaptured exosomes (**Figure 2B**). No gold deposition (namely, anti-SARS-CoV-2-S RBD labeling) was detectable in HD micrographs (**Figure 2C** upper panel), whereas an increased level of gold deposition was evident on the ringlike surface of exosomes of MILD compared to SEVERE patient samples (**Figure 2C** middle and lower panel). To better visualize SARS-CoV-2-S-derived peptides at the single-molecule level and define their spatial localization on the surface of exosomes, we performed single-molecule localization microscopy *via* TIRF/Direct-STORM with stochastic blinking reconstruction, as previously reported (29). Given the high density of blinking fluorophores needed for this approach, the experiment was performed only on MILD patient exosomes. Single-molecule imaging of MILD patient exosomes revealed that SARS-CoV-2-S-derived fragments were widespread and highly abundant across the entire exosomal surface (**Figures 2D–F**). Moreover, we also performed 3D-STORM on best-emitting samples in order to retrieve within the TIRF plan a correct volumetric localization of blinking molecules. Such analysis further confirmed the localization of RBD at the level of the exosome surface, with no signal localizing within the inner vesicle compartment (**Figure 2G** and **Supplementary Video 1**).

### Exosomes of MILD COVID-19 Patients Induce CD4^+^ T-Cell Activation More Efficiently Than Those of SEVERE Patients

**Figure 3:**
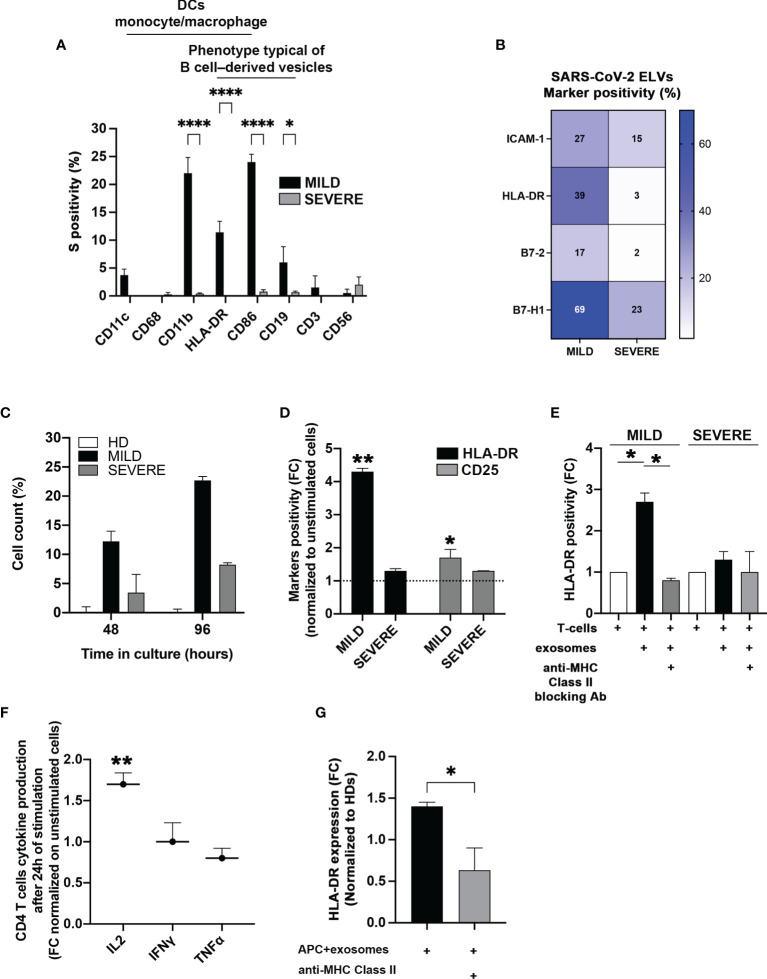
Exosomes recovered from the plasma of MILD COVID-19 patients stimulate CD4^+^ T-cell immune responses. **(A)** Cytofluorimetric analysis of immune cell markers. SARS-CoV-2-S^+^ exosomes recovered from the plasma of COVID-19 patients and immunocaptured with anti-CD63/CD9 latex beads were analyzed by flow cytometry with the indicated surface markers (Two-way ANOVA with Tukey test, *p < 0.03; ****p < 0.0001). **(B)** Cytofluorimetric analysis of costimulatory molecules and ICAM-1 on patient-recovered exosomes. Exosomes recovered from patients (N = 6) were captured with anti-CD63 beads and gated for CD9 positivity. The heatmap shows expression levels of the indicated markers based on a color scale from white (no expression) to blue (highest expression). **(C)** Stimulation of CD4^+^ T cells. Coculture of sorted CD4^+^ T cells with exosomes isolated from the plasma of MILD and SEVERE patients or HDs for up 96 h. Trypan blue was used to discriminate between live and dead cells. Results are representative of three independent experiments performed in triplicate. **(D, E)** Activation of CD4^+^ T cells. **(D)** The stimulatory effect of exosomes recovered from plasma of COVID-19 patients on CD4^+^ T cells was measured by flow cytometry, detecting the expression of HLA-DR (black bars) and CD25 (gray bars) after 2 days of culture. Unstimulated cells were used as a threshold to calculate marker positivity (dotted line). **(E)** The same assay as in panel **(C)** was conducted with or without HLA-DR blocking antibodies (t-test, *p < 0.05, **p < 0.01 and one-way ANOVA with Tukey test, *p < 0.03). **(F)** Cytokine analysis. Production of the indicated cytokines by CD4^+^ T cells was measured after 24 h of incubation with exosomes. **(G)** Flow cytometry analysis of HLA-DR induction by exosomes. The graphs show the expression of HLA-DR on isolated CD4^+^ T cells following incubation with matched monocytes pulsed with exosomes recovered from MILD COVID-19 patients. HLA-DR blockade was performed by treating the cells with anti-HLA-DR antibodies (Double-tailed t-test, *p < 0.05, **p < 0.01). HD, Healthy donor; S/Co, Signal/Control; RFI, Relative Fluorescence Intensity; S, Spike; rec, recombinant; Hsp70, Heat shock protein 70; TSG101, tumour susceptibility gene 101; ApoA, Apolipoprotein A; AF488, Alexa Fluor 488; APC, Allophycocyanin; Ctrl, Control.

The data obtained so far suggested that the higher abundance of SARS-CoV-2-S^+^ exosomes could be somewhat beneficial to MILD patients and contribute to their better outcome during viral infection. Among various possibilities, we hypothesized that exosomes could contribute to the adaptive immune response against SARS-CoV-2 infection by possibly exposing SARS-CoV-2-S-derived particles. To verify this assumption, at first, we analyzed the origin of SARS-CoV-2-S^+^ exosomes. Since all exosomes contain proteins derived from the parent cell and can partly maintain parent cell functionality, we characterized exosome origin using antibodies against major immune cell surface receptors. We found that SARS-CoV-2-S^+^ exosomes of MILD COVID-19 patients were mostly of B cell, dendritic cell, and monocyte/macrophage origin, as they displayed B-cell marker CD19, integrin CD11b, costimulatory molecule CD86, and MHC-class II HLA-DR (**Figure 3A**). This result indicated that most MILD patient exosomes containing SARS-CoV-2-S fragments are derived from antigen-presenting cells (APCs) and could possibly maintain antigen-presenting functions. Since proper antigen-presenting capability requires the presence of both adhesion molecules and costimulatory factors, we quantified the levels of adhesion factor Intercellular Adhesion Molecule 1 (ICAM-1) and of costimulatory molecules Human Leukocyte Antigen-DR isotype (HLA-DR), B7-2, and B7-H1 (30–32) on the surface of patient-recovered exosomes. We found that all MILD patient exosomes were in general more enriched in all the markers analyzed (**Figure 3B**). These data suggested that SARS-CoV-2-S^+^ exosomes recovered from MILD COVID-19 patients could act *per se* as antigen-presenting vehicles and modulate antigen-specific T-cell responses. To verify this hypothesis, at first, we incubated *in vitro* exosomes of COVID-19 patients and HDs with autologous CD4^+^ T cells and analyzed cell proliferation. We found that COVID-19 patient-recovered exosomes were capable of stimulating CD4^+^ T-cell growth, while HD-recovered exosomes were not (**Figure 3C**). Most interestingly, exosomes of MILD COVID-19 patients were more efficient in stimulating CD4^+^ T-cell growth compared to those of SEVERE COVID-19 patients (**Figure 3C**). To assess whether exosomes of MILD COVID-19 patients induced T-cell growth by actual T-cell activation, we tested their capability to modulate the expression of middle, CD25, and late, HLA-DR, T-lymphocyte activation markers cocultured in matched CD4^+^ T cells. We found that both markers were actually drastically upregulated only in the samples cocultured with exosomes of MILD COVID-19 patients (**Figure 3D**), suggesting that T-cell proliferation was indeed a consequence of efficient T-cell activation triggered by exosomes. Comparable results were obtained when we repeated the assay using allogeneic T cells derived from HDs, thereby excluding the possibility that the lower immunostimulatory activity of SEVERE patient exosomes could be due to dysfunctional T cells (data not shown).

Next, we checked whether antigen presentation was driving exosome-dependent T-cell activation. We replicated the previous experiment but blocking MHC-II with specific antibodies. We found that MHC-II blockade significantly reduced exosome-dependent T-cell activation (**Figure 3E**), confirming that an antigen-presenting activity of MILD COVID-19 patient exosomes was the actual driving force of CD4^+^ T-cell activation. This evidence was further confirmed by the fact that MILD patient exosomes stimulated CD4^+^ T cells to produce high levels of IL-2, a cytokine whose secretion is known to be triggered early during antigen presentation (**Figure 3F**). Interestingly, two other cytokines secreted during antigen presentation, interferon (IFN)γ and tumor necrosis factor (TNF)α, were not enriched possibly due to their different induction kinetics (**Figure 3F**).

Next, we investigated if MILD COVID-19 patient-recovered exosomes were capable of favoring CD4^+^ T-cell activation driven by other APC sources. We checked if MILD COVID-19 patient-recovered exosomes could enhance CD4^+^ T-cell activation. Coculturing MILD COVID-19 patient exosomes with CD4^+^ T cells and autologous monocytes as primary APC source caused a significant increase in CD4^+^ T-cell HLA-DR protein levels (**Figure 3G**), an effect that was totally abolished when we blocked MHC-II, confirming the requirement of cross-presentation for efficient CD4^+^ T-cell activation and indicating a peculiar antigen cross-presentation activity of MILD COVID-19 exosomes.

Our findings suggest that MILD COVID-19 patient-recovered exosomes are capable of favoring CD4^+^ T-cell activation by functioning as an antigen-presenting source and by promoting T-cell activation.

### The Protein Repertoire of Exosomes Recovered From The Plasma of MILD COVID-19 Patients Correlates With a Signature of Immune Response Enhancement

**Figure 4:**
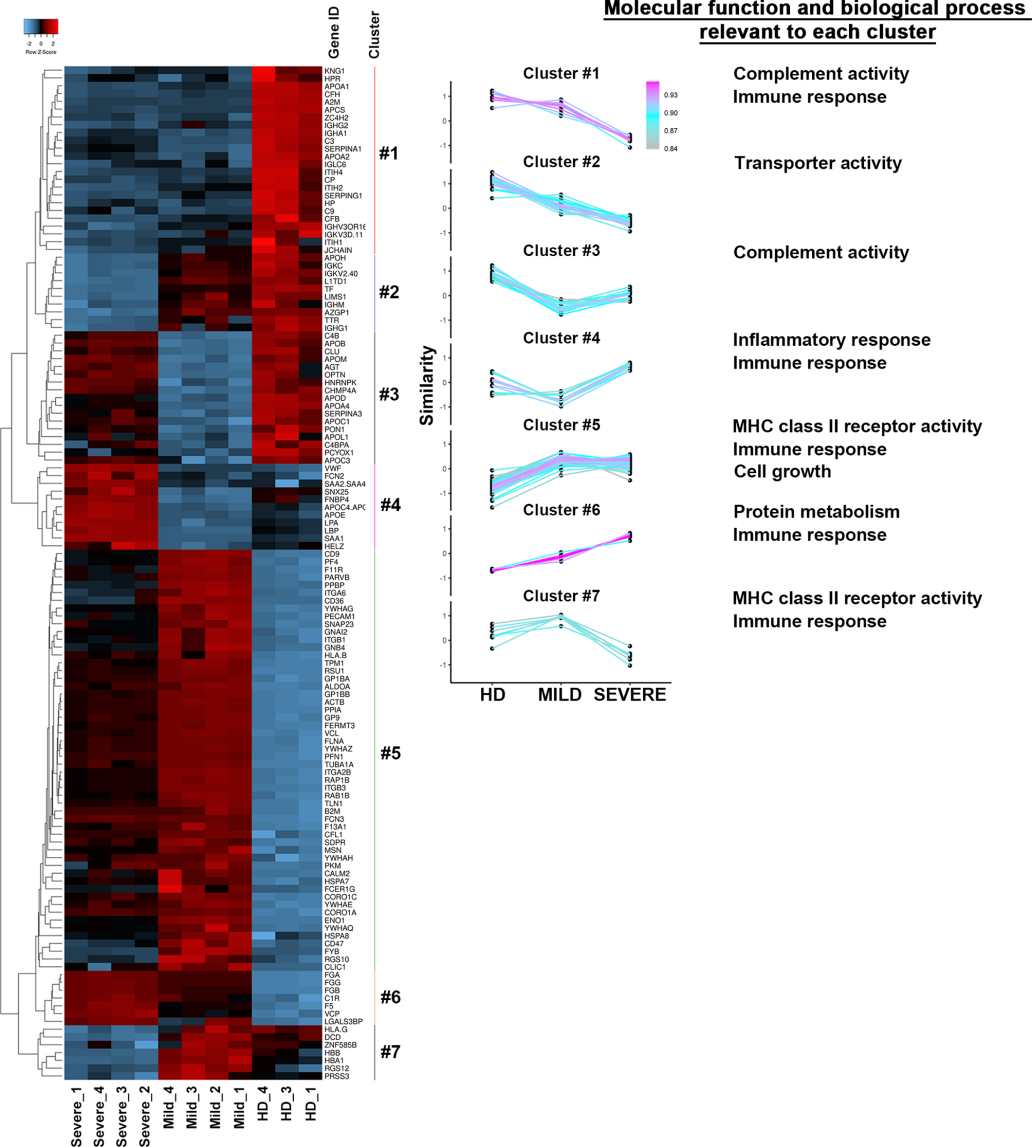
The protein repertories of exosomes recovered from COVID-19 patients and HDs are drastically different. (Left) Heatmap of the 130 proteins identified in the exosomes of either MILD or SEVERE COVID-19 patients and HDs. Color bars represent Z-score changes from −2 to 2. Hierarchical cluster analysis between replicates using the Euclidean’s method with average linkage distance showing a clear group differentiation according to similarity. (Right) The list of different clusters was analyzed in terms of Molecular function and Biological process using the FUNRICH software. The most significant results (calculated with the Hypergeometric test; Bonferroni correction) are annotated on the right in the figure. HD, Healthy donor; S/Co, Signal/Control; RFI, Relative Fluorescence Intensity; S, Spike; rec, recombinant; Hsp70, Heat shock protein 70; TSG101, tumour susceptibility gene 101; ApoA, Apolipoprotein A; AF488, Alexa Fluor 488; APC, Allophycocyanin; Ctrl, Control.

With the intent to corroborate our results and better define the functional roles of COVID-19 patient-recovered exosomes, we defined their proteomes. For each patient class (MILD and SEVERE), we selected the 4 exosome samples with the highest SARS-CoV-2-S positivity (indicated by an asterisk in **Supplementary Table S1**), while for HDs, exosome samples were selected randomly. Mass spectrometry was performed on whole exosome protein content. Comparing the proteomes of the three sets of samples, we identified 130 differentially abundant proteins (**Supplementary Table S2**), 92% of which were previously annotated in public EV databases [EXOCARTA (33)] as EV/exosome components, confirming the efficacy of our exosome purification. As expected, and in line by previously reported studies regarding conventional exosome purification methods (34, 35), highly abundant apolipoproteins were detected in all three sample types analyzed (**Supplementary Table S2**) but did not interfere with downstream analyses. Pairwise correlation analysis of protein contents clearly revealed that samples of the same class displayed a high internal correlation mirrored by lower correlation with samples of other classes (**Figure S4A**), indicating a high homogeneity among exosomes recovered from different donors. This result was further confirmed by hierarchical clustering that, by gathering the 130 proteins into 7 groups, clearly separated MILD COVID-19-, SEVERE COVID-19-, and HD-recovered exosomes (**Figure 4**, left). To assess the characteristics of each cluster found, we carried out a comparative analysis using FunRich (36). Our results revealed that the clusters linked to HD exosomes correlated with complement activity and immune response activation, those linked to SEVERE COVID-19 patient exosomes correlated with immune/inflammatory responses and protein metabolism, while those mainly related to MILD COVID-19 patient exosomes were related to immune response, cell growth, signal transduction, and MHC class II receptor functionality (**Figure 4**, right), well confirming our *in vitro* results that depicted MILD COVID-19 patient exosomes as strong stimulators of CD4^+^ T-cell activation and growth.

**Figure 5:**
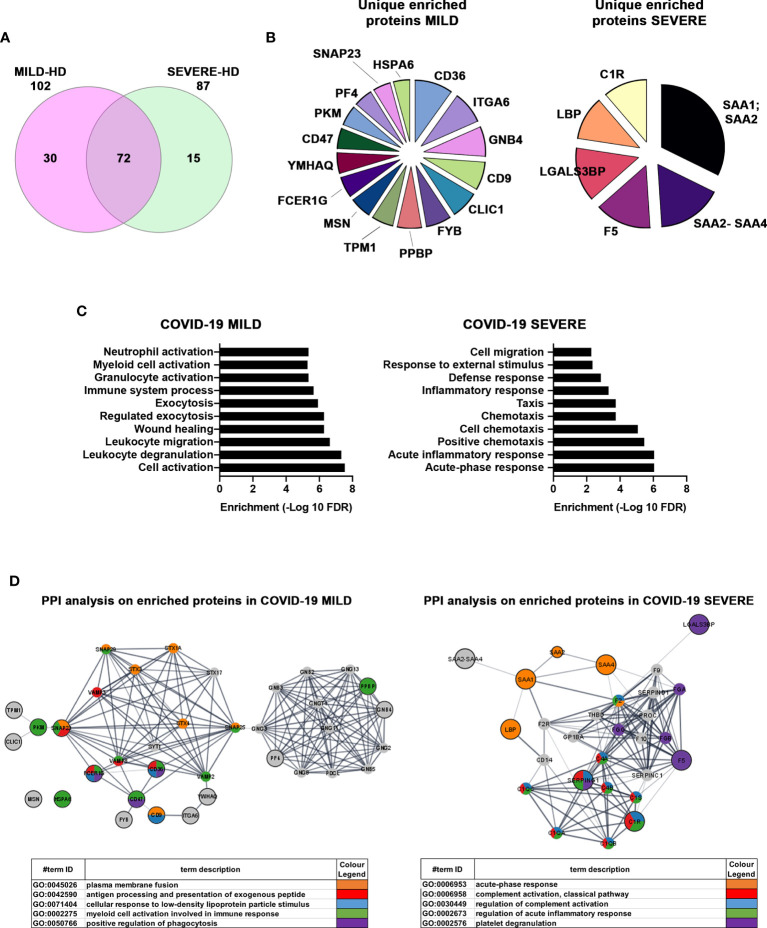
Proteins enriched in exosomes of MILD COVID-19 patients are mainly involved in the immune response, while those enriched in the exosomes of SEVERE COVID-19 patients correlate with stress responses and acute inflammation. **(A)** Venn diagram representing the differentially abundant proteins residing in the exosomes of MILD (pink) and SEVERE (green) COVID-19 patients compared to the HD control group [cutoff value: Log fold change >3 and <-0.5; p value (t test) < 0.05]. Here, 102 and 87 proteins were identified as differentially expressed in MILD and SEVERE patients, respectively. The overlap among the two groups of COVID-19-infected patients shows that 72 proteins are present in both samples, while 45 are differentially abundant. **(B)** Pie charts of unique and enriched proteins derived from the exosomes of MILD (16 proteins) and SEVERE (6 proteins) COVID-19 patients. **(C)** The detected proteins were analyzed by GO term enrichment analysis (ShinyGO v0.61) using a p-value cutoff of (FDR) <0.01. Significantly enriched categories are shown. **(D)** Network interaction analysis for the enriched proteins related to exosomes of COVID-19 patients. Known and predicted protein–protein interactions (PPIs) were extracted from the STRING database (Version 11.0). Proteins are depicted as nodes, and the biological relationships between nodes are represented as edges (lines). The inputs are represented in gray, while other colors indicate the different pathways to which the inputs belong to. Tables summarize the GO enrichment analysis for biological processes. HD, Healthy donor; S/Co, Signal/Control; RFI, Relative Fluorescence Intensity; S, Spike; rec, recombinant; Hsp70, Heat shock protein 70; TSG101, tumour susceptibility gene 101; ApoA, Apolipoprotein A; AF488, Alexa Fluor 488; APC, Allophycocyanin; Ctrl, Control.

Next, we focused on differentially abundant proteins. We identified 102 proteins differentially present in MILD COVID-19- vs. HD-recovered exosomes and 87 proteins differentially abundant in SEVERE COVID-19- vs. HD-recovered exosomes. Here, 72 proteins were shared between the two groups, while a total of 45 proteins were specific for one group or the other (**Figure 5A** and **Figure S4B**). By focusing on the latter 45 proteins, we selected the 22 with the highest enrichment. These included 16 proteins for the MILD and 6 for the SEVERE patient group (**Figure 5B**). GO term analysis indicated that the proteins recovered from MILD COVID-19 patient exosomes were mainly involved in immune cell activation, while those recovered from SEVERE COVID-19 patient exosomes were mainly involved in stress and inflammatory responses (**Figure 5C**). Such results were also confirmed by interrogating the STRING (37) database. Exosomes recovered from MILD COVID-19 patients showed an enrichment in proteins involved in pathways related to antigen processing, presentation of exogenous peptides (FDR <1.56E-07), and myeloid cell activation (FDR <6.65E-08) (**Figure 5D**, left), while those recovered from SEVERE COVID-19 patients showed an enrichment in proteins involved in pathways regulating acute inflammatory responses (FDR <3.58E-13) and complement activation (FDR <3.07E-14) (**Figure 5D**, right), further strengthening the notion that MILD and SEVERE patient exosomes are drastically different and possess different immunomodulatory functions, the first mainly involved in immune cell activation and the second principally involved in inflammation.

## Discussion

Exosomes and other types of EVs play a number of critical roles in cell-to-cell communication. Their composition and biological activities change depending on their origin and can be drastically modified by bacterial, fungal, and viral infections (38). It has been reported that EVs may incorporate pathogen proteins and/or fragments of viral RNA from infected cells in order to shuttle the material to target cells, an event which plays important roles in viral infection responses (39–41). In addition, there is evidence that viruses can use endocytic routes to enter uninfected cells and/or hijack EV secretory pathways to exit infected cells, indicating that EVs and viruses share common cell entry and biogenesis mechanisms (42, 43). Through the years, several studies analyzed the role of EVs and exosomes in viral infection, in particular in HIV, hepatitis C virus (HCV), and SARS pathologies (39, 40). Recently, a few works also characterized EVs of COVID-19 patients (44, 45), but an actual role in adaptive immunity was never assessed.

Here, we characterized the protein composition of exosomes of COVID-19 patients who experienced MILD or SEVERE symptoms, collecting samples around 14 days after diagnosis.

Unfortunately, for each sample analyzed, we had availability to a very limited amount of material, and this posed some methodological restrictions. For instance, we could not perform conventional collection of exosomes by ultracentrifugation. Exosomes were instead isolated and visualized by adopting different commercial kits and experimental approaches more suitable for low sample volumes but still reliable and well accepted by the scientific community (e.g., EXOTEST and ExoView).

We found that MILD COVID-19 patient exosomes had higher levels of the MHC class II receptor, which is responsible for antigen presentation to CD4^+^ T helper cells and high levels of both CD11b, a differentiation marker for cells of the myeloid–monocytic lineage (46, 47), and CD86, a type I transmembrane protein originally identified as a CD28/CTLA-4 ligand, which are both associated with T-cell activation (48). Strikingly, by using different approaches, we found that exosomes of MILD patients bear on their surface detectable SARS-CoV-2-S-derived fragments that could derive from either an active phagocytic activity or a transient viral infection of paternal cells. The isolation technique we used, based on antibody purification of the vesicles, excluded the possibility that SARS-CoV-2-S-derived fragments could originate from virus particle contaminants co-purifying with our exosomes.

MILD COVID-19 patient exosomes are able to activate both autologous and heterologous CD4^+^ T helper cells and induce IL-2 secretion *in vitro*, suggesting that *in vivo*, they could enhance the immune responses elicited against SARS-CoV-2 antigens, thereby possibly contributing to a better outcome or more rapid resolution of the infection. The fact that this ability was observed in both matched or unmatched PBMC samples excludes any potential bias given by T cell dysfunctionality in SEVERE COVID-19 patients and could suggest that the immunomodulatory effect is due at least in part to the presence of SARS-CoV-2-S-derived fragments exposed on the exosomal surface.

By characterizing patient-recovered exosomes, we generally observed that those of SEVERE patients harbored proteins involved in metabolism, inflammation, and stress responses, while those recovered from MILD patients showed an enrichment in proteins involved in immune activation, effector activity, and migration/chemotaxis, possibly reflecting a more efficient functioning of the immune system. In particular, our results identified specific features of MILD and SEVERE COVID-19 patient exosomes. Here, 16 proteins were unequivocally associated with MILD COVID-19 patient exosomes, while 6 were found enriched exclusively in those of SEVERE COVID-19 patients (**Figure 5B**). Among the enriched MILD patient exosome proteins we found are the following: CLIC1, CD9, FYB, CD36, CD47, and SNAP23, which are involved in antigen processing and cross-presentation (49–53) and trigger T-cell activation; PF4 and PPBP, which act as chemoattractants and activate, respectively, neutrophils and monocytes (54); MSN, which acts on both T- and B-cell homeostasis by regulating lymphocyte egress from lymphoid organs (55); and ITGA6, which negatively impacts virus transcription (56). Exosomes of patients with SEVERE disease were instead enriched in complement factors, members of the coagulation system, inflammation modulators, and regulators of IL-6 pro-inflammatory signaling. Specifically, we found C1R, which is known to initiate complement activation (57, 58) and was shown to play a major role in acute and chronic inflammation, endothelial cell dysfunction, thrombus formation, and intravascular coagulation in SARS-CoV-2 patients (59); SAA2-SAA4 and SAA1-SAA2, which are markers of inflammatory response and tissue injury (60) and are induced by IL-6 (61); LGALS3BP, a pro-inflammatory factor (62) that is known to induce IL-6 expression (63); and LBP, whose upregulation contributes to inflammation (64) and correlates with immune response dysregulation in both pneumonia (65) and SARS-CoV-2 patients (64).

Collectively, our study confirms that plasma-recovered exosomes reproduce the molecular patterns of their cells of origin and reflect the different pathological states of COVID-19 patients. In agreement with published data (45, 66), our proteomic analysis of patient-recovered exosomes identified several molecules involved in immune responses, inflammation, and activation of both coagulation and complement pathways, which are the main mechanisms of COVID-19-associated tissue damage. We also highlighted that exosomes are peculiar indicators of the functional state of patients’ immune cells, which are generally found to be better performing in individuals with MILD symptoms. This specific feature provides the rationale for future studies on alternative exosome-based preventive or prophylactic approaches to treat SARS-CoV-2 infection.

## Data Availability Statement

The mass spectrometry proteomics data have been deposited to the ProteomeXchange Consortium *via* the PRIDE partner repository with the dataset identifier PXD029007.

## Ethics Statement

The study was approved by the Institutional Review Board Milano Area 2 (#331_2020). The patients/participants provided their written informed consent to participate in this study.

## Author Contributions

Conceptualization, EP. Investigation, EP, NM, CC, SS, PG, AC, FC, GC, and AF. Writing—original draft, EP, NM, and RG. Resources, AB, RU, AL, DM, AM, SAI, FB, and AGor. Formal analysis, AGob and MB. Supervision, SAb, RDF, SB, and RG. Funding acquisition, RG. All authors reviewed and edited the drafts. All authors contributed to the article and approved the submitted version.

## Funding

This research was supported by the project COiMMUNITY (ID 1842163) to RG funded by Regione Lombardia, funded under POR FESR 2014‐2020 resources, and by the project “END-COVID” (B/2020/0076) to AGor funded by Intesa San Paolo “Fondo di beneficenza ed opere di carattere sociale e culturale,” and by an unrestricted grant from Fondazione “Romeo ed Enrica Invernizzi”.

## Conflict of Interest

The authors declare that the research was conducted in the absence of any commercial or financial relationships that could be construed as a potential conflict of interest.

## Publisher’s Note

All claims expressed in this article are solely those of the authors and do not necessarily represent those of their affiliated organizations, or those of the publisher, the editors and the reviewers. Any product that may be evaluated in this article, or claim that may be made by its manufacturer, is not guaranteed or endorsed by the publisher.

## Supplementary Material

Supplementary Video 1 (linked to **Figure 2G**). Video rendering of volumetric 3D image from stochastically reconstructed blinking single molecules on 3D STORM TIRF plan, executed on one representative EV, immunolabeled for RBD (with secondary labeling in AlexaFluor-647) from MILD COVID-19 patient. Video projections clearly highlight the majority of RBD-labeled molecules on the surface of the EV, with an inner part devoid of RBD signal, thus suggesting a specific 3D surface localization of RBD protein on plasma-derived EVs. Localized molecules are shown in pseudo-color 16-ramp scale to better perceive differences in signal intensities.
