# Supplementary material for: Exosomes Recovered From the Plasma of COVID-19 Patients Expose SARS-CoV-2 Spike-Derived Fragments and Contribute to the Adaptive Immune Response
Source: Front Immunol. 2022 Jan 17;12:785941. doi: 10.3389/fimmu.2021.785941 (PMC8801440; doi:10.3389/fimmu.2021.785941)
Supplement: Supplementary file 2 [file DataSheet_1.pdf]

Supplementary Material

Supplementary Figures and Tables

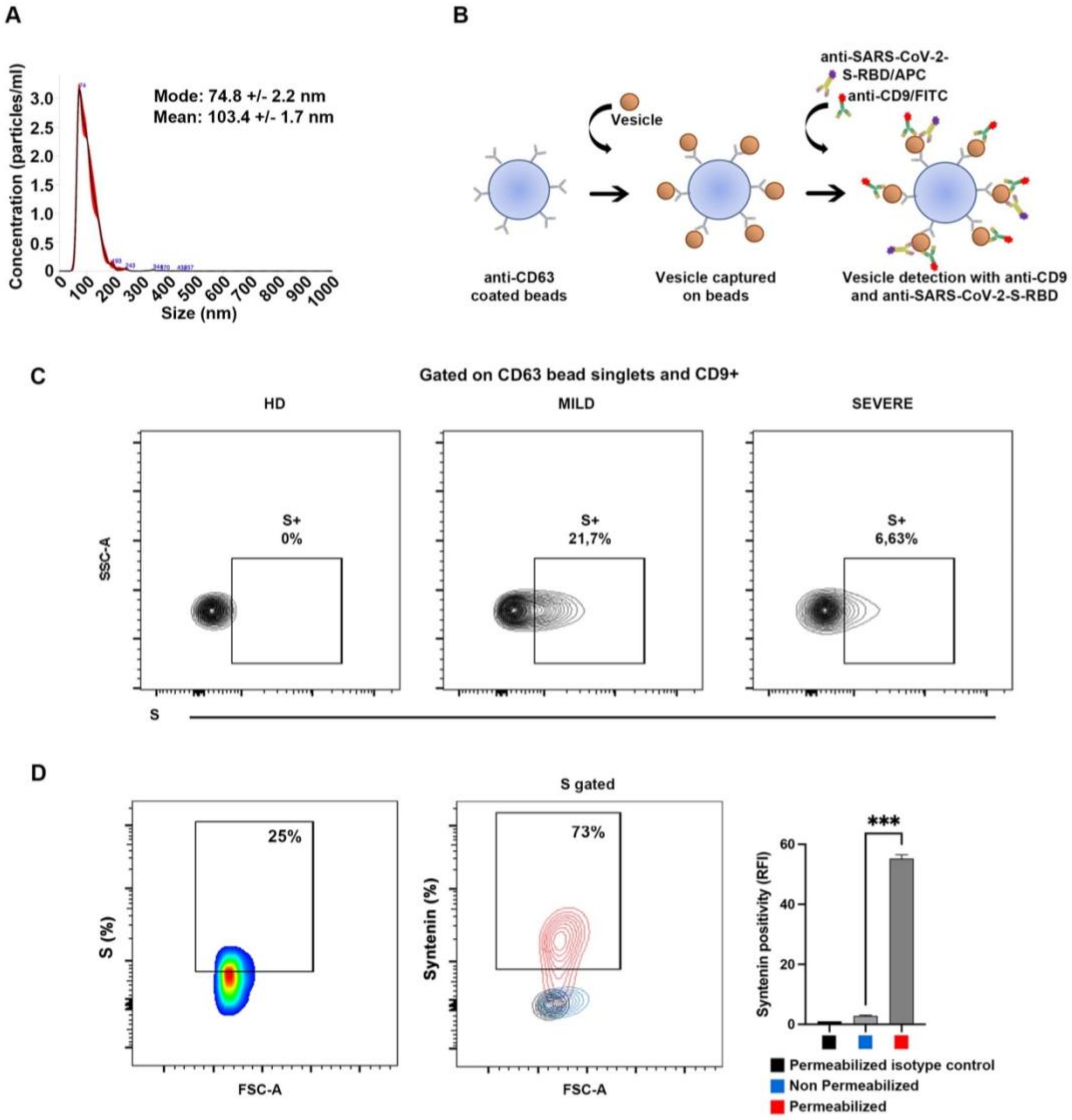

Supplementary Figure 1. Characterization of plasma-recovered-exosomes from COVID-19 patients and flow cytometry detection of immunocaptured SARS-CoV-2-S+ exosomes.

A) Representative histogram of particle concentration and size distribution of plasma-recovered exosomes measured by nanoparticle tracking analysis (NTA). B) Scheme of ELV immunocapturing and subsequent staining. Latex beads were coated with anti-CD63 antibodies and incubated with

plasma-derived ELVs. Captured ELVs were then stained with anti-CD9 and anti-SARS-CoV-2-S-RBD antibodies and subjected to flow cytometry. C) Representative flow cytometry contour plots showing $CD9^+$ ELVs stained with anti SARS-CoV-2-S-RBD antibodies. Images are representative of three independent experiments. D) Representative flow cytometry analysis showing integrity of SARS-CoV-2-S^+ exosomes. Exosomes were either permeabilized (red) or left untreated (blue) and syntenin levels were assessed. The graph on the right shows syntenin positivity reported in RFI (value vs isotype control), and confirms its detection only after permeabilization of exosomes.

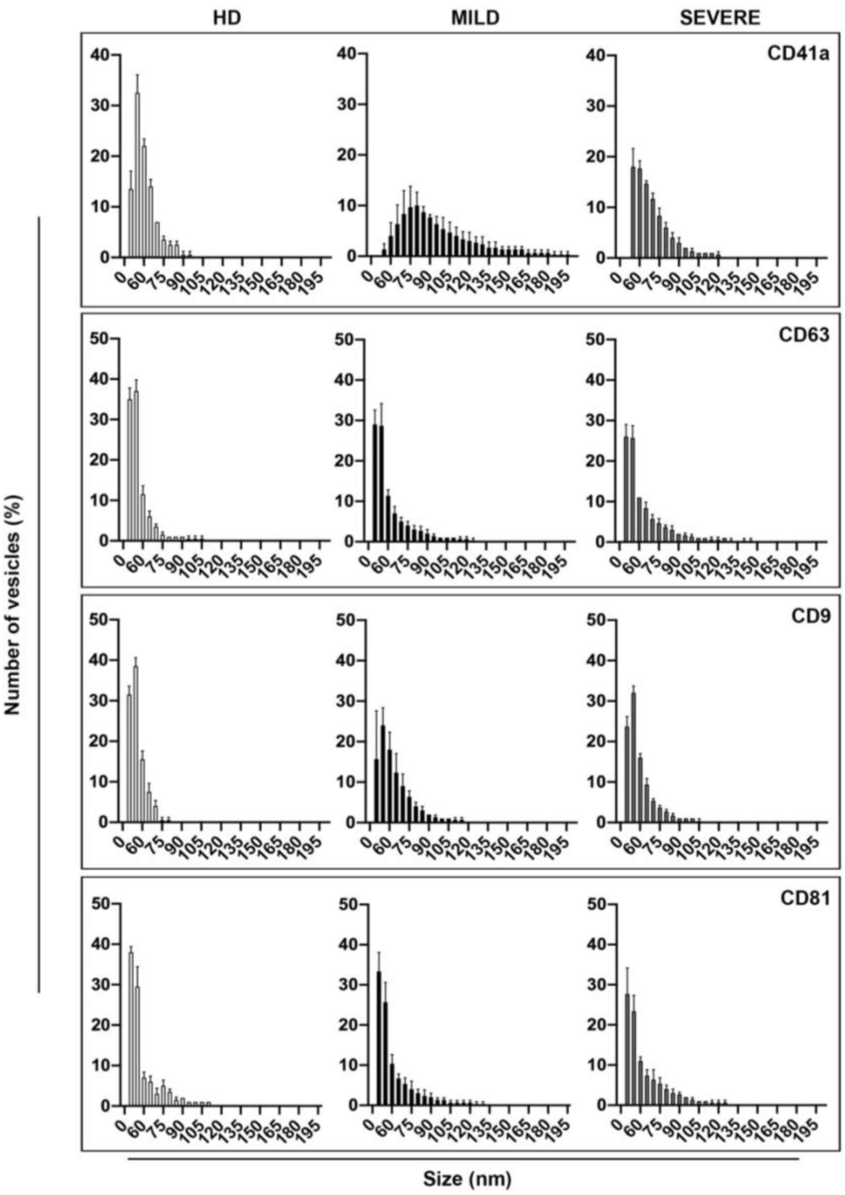

Supplementary Figure 2. ELVs derived from the plasma of MILD and SEVERE COVID-19 patients are larger than those derived from HDs.

Interferometric measurement of plasma-derived ELVs of HDs, MILD and SEVERE COVID-19 patients were obtained using the ExoView platform. CD9, CD63, CD81 and CD41a -positive ELVs were immuno-captured on a microarray chip and imaged. Images were then analyzed to enumerate and size the ELVs bound to each antibody spot on the chip. Vesicles were measured in a 50-200 nm diameter range.

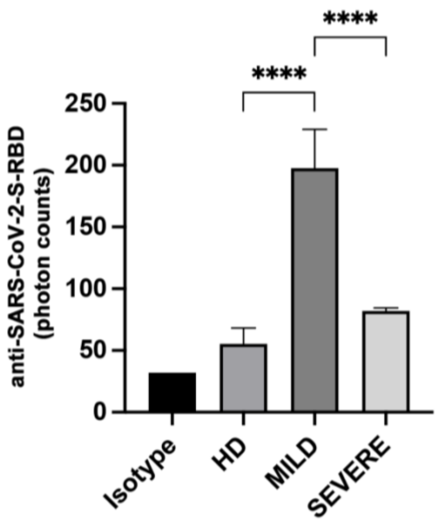

Supplementary Figure 3. Labeling quantification of SARS-CoV-2-S^{+} ELVs.

Detection of SARS-CoV-2-S in bead-captured ELVs using STED microscopy as described in main Figure 2D. Quantification of immunolabelled anti-SARS-CoV-2-S-RBD*STAR-RED photon counts was performed on n=45, n=53, n=51 beads from HD, MILD and SEVERE samples respectively (n=2 independent biological replicates). Results were compared to the Isotype*STAR-RED signal. Bar-charts represent means ± SEM for each sample per condition (MILD samples =197±31 photons, SEVERE samples =82±2 photons, HD samples= 55±13 photon, unspecific isotype labelling = 32). (Paired T-test, ****; $p < 0.0001$).

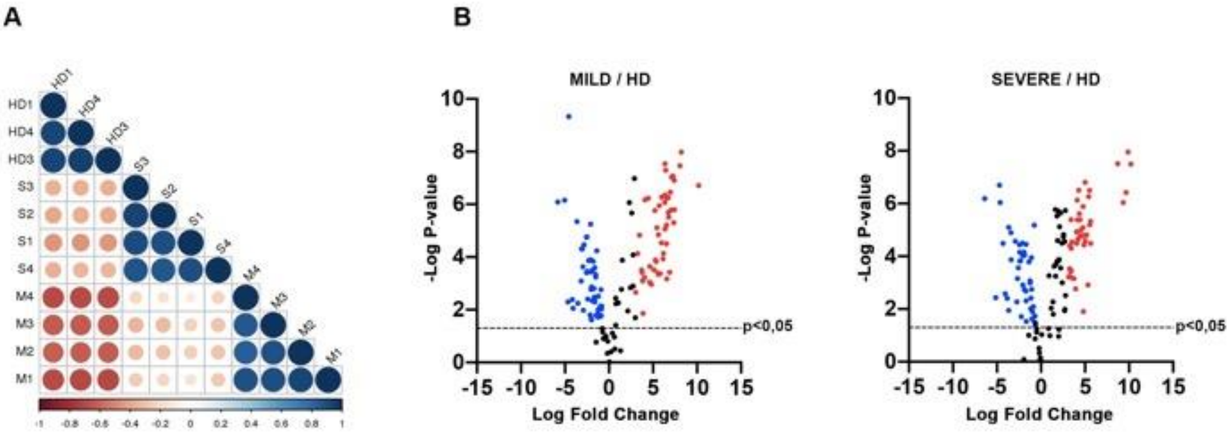

Supplementary Figure 4. The protein composition of plasma derived ELVs distinguishes between COVID-19 patients and HDs

A) Pairwise similarity matrix between replicate ELV samples based on Spearman's correlations. MILD: M1-4, SEVERE: S1-4 and HDs: 1,4,3. Correlation ranges from zero (no correlation; red) to one (perfect correlation; blue). B) Volcano plots showing differentially abundant proteins between either MILD (left panel) or SEVERE (right panel) COVID-19 patients and HD control ELVs. The red dots represent overexpressed proteins (51 proteins for MILD and 41 for SEVERE) while the blue dots represent downregulated proteins (51 proteins for MILD and 46 for SEVERE). Cutoffs were set at 0.05 for p-values (dashed horizontal line) and < -0.5 or > 3 for fold changes (FCs).

Supplementary Table 1. Clinical Characteristics of COVID-19 patients.

ID PATIENT	SEX	AGE	Sampling day (from positive swab)	CLASS	SUB-CLASS	Comorbility	Respiratory support therapy	Pneumonia	Symptoms
PMI-0028	F	33	T19	Mild	acute	none	NO	NO	Fever, cough, pharyngodynia, ageusia, anosmia
PMI-0029*	F	22	T16	Mild	acute	Asthma	NO	NO	Fever, cough, headache
PMI-0032*	F	29	T14	Mild	acute	none	NO	NO	Fever, pharyngodynia
PMI-0031*	M	77	T15	Mild	acute	none	NO	NO	Fevere, cough, diarrhea
PMI-0003*	M	31	T16	Mild	acute	none	NO	NO	coryza
PMI-0022	M	41	T16	Mild	acute	none	NO	NO	none
PMI-0002	M	27	T16	Mild	acute	Asthma	NO	NO	Fever, cough
PMI-0009	M	47	T14	Mild	acute	none	NO	NO	Fever, cough
PMI-0040	F	77	T16	Mild	acute	none	NO	NO	coryza
PMI-0027	F	27	T18	Mild	acute	none	NO	NO	none
PMI-0001	F	55	T6	Severe	acute	none	yes	yes	Fever, cough, dyspenea, sputum
PMI-0004*	F	37	T14	Severe	acute	GERD, obesity	yes	yes	Fever, asthenia
PMI-0005*	M	60	T17	Severe	acute	Hypertension	yes	yes	Fever, dyspnea, asthenia, loss of smell and taste
PMI-0033*	M	61	T14	Severe	acute	Hypertension	yes	yes	Fever, cough, dyspnea, respiratory failure
PMI-0034*	F	68	T14	Severe	acute	Hyperthyroidism, Budd-Chiari syndrome, polycythemia vera, myelofibrosis	yes	yes	Fever, dyspnea
PMI-0039	M	65	T14	Severe	acute	Hypertension	yes	yes	Fever, dyspnea
PMI-0052	M	72	T14	Severe	acute	none	yes	yes	Fever, cough, dyspnea
PMI-0044	M	64	T14	Severe	acute	Hypertension	yes	yes	Fever, dyspnea
PMI-0010	F	78	T2	Severe	acute	Hypertension, diabetes	yes	yes	Fever, cough, diarrhea, vomiting
PMI-0045	M	83	T14	Severe	acute	Ischemic heart disease	yes	yes	Fever, cough, dyspnea

Supplementary Table 2. Proteomic data from plasma-derived exosome-like veiscles of healthy donors and MILD and SEVERE COVID-19 patients.

Gene names	Protein name	S_1	S_4	S_3	S_2	M_4	M_3	M_2	M_1	HD_4	HD_3	HD_1	CLU
KNG1	Kininogen-1	-1,02	-0,79	-0,37	-0,21	-0,24	-0,26	-0,33	-0,70	2,53	0,56	0,84	1
HPR	Haptoglobin-related protein	-0,75	0,00	-0,05	-0,42	-1,30	-0,34	-0,06	-0,86	2,35	1,00	0,43	1
APOA1	Apolipoprotein A1	-0,84	-0,61	-0,67	-0,68	-0,44	-0,25	-0,43	-0,66	1,68	1,66	1,25	1
CFH	Complement Factor H	-0,79	-0,59	-0,61	-0,73	-0,59	-0,37	-0,47	-0,48	1,61	1,58	1,44	1
A2M	Alpha-2-Macroglobulin	-0,73	-0,52	-0,52	-0,55	-0,66	-0,55	-0,55	-0,57	1,75	1,55	1,35	1
APCS	Serum amyloid P-component	-0,51	-0,42	-0,57	-0,64	-0,44	-0,46	-0,43	-0,44	1,79	1,73	1,76	1
ZC4H2	Zinc finger C4H2 domain-containing protein	-0,63	-0,58	-0,45	-0,87	-0,66	-0,44	-0,45	-1,08	1,52	1,37	0,98	1
IGHG2	Immunoglobulin Heavy Constant Gamma 2	-0,99	-0,84	-0,62	-0,94	-0,36	0,24	-0,31	-0,53	1,62	1,60	1,14	1
IGHA1	Ig alpha-1 chain Immunoglobulin Heavy Constant Alpha 1C region	-0,56	-0,24	-0,26	-0,38	-0,74	-0,98	-0,56	-0,81	1,70	1,55	1,29	1
C3	Complement C3	-0,40	-0,20	-0,33	-0,24	-0,89	-0,78	-0,76	-0,89	1,79	1,47	1,22	1
APOA2	Apolipoprotein A-II	-0,39	-0,20	-0,29	-0,45	-0,97	-0,73	-0,26	-1,12	1,67	1,62	1,13	1
SERPINA1	Serpin Family A Member 1	-0,32	-0,05	-0,03	-0,26	-0,78	-0,84	-0,87	-1,13	1,86	1,41	1,01	1
IGLC6;IGLC7	Immunoglobulin Lambda Constant 6;7	-0,93	-0,87	-0,26	-0,22	-0,15	-0,79	-0,02	-1,02	1,76	1,68	0,81	1
ITIH4	Inter-alpha-trypsin inhibitor heavy chain 4	-0,85	-0,66	-0,49	-0,53	-0,36	-0,52	-0,41	-0,49	1,91	1,91	0,50	1
CP	Ceruloplasmin	-0,55	-0,49	-0,82	-0,64	-0,41	-0,63	-0,33	-0,44	1,98	1,83	0,48	1

**ITIH2**	**Inter-Alpha-Trypsin Inhibitor Heavy Chain 2**	-0,95	-0,82	-0,87	-0,68	-0,28	-0,33	-0,10	-0,11	1,86	1,83	0,44	1
**SERPING1**	**Plasma protease C1 inhibitor**	-0,86	-0,64	-0,81	-0,93	-0,57	-0,21	-0,50	-0,43	1,79	1,36	0,56	1
**HP**	**Haptoglobin**	-0,83	-0,38	-0,22	-0,48	-0,98	-0,31	-0,53	-0,59	1,94	1,76	0,62	1
**C9**	**Complement component C9**	-0,39	0,02	-0,95	-0,28	-0,88	-0,71	-0,48	-0,50	1,97	1,73	0,47	1
**CFB**	**Complement factor B**	-0,82	-0,73	-0,67	-0,80	-0,46	-0,16	-0,18	-0,29	1,15	2,39	0,57	1
**IGHV3OR16-9;IGHV3-72;IGHV3-73;IGHV3-15**	**Immunoglobulin Heavy Variable**	-0,64	-1,36	-1,16	-0,88	-0,36	-0,47	-0,46	-0,15	0,96	0,97	1,23	1
**IGKV3D-11;IGKV3D-15;IGKV3OR2-268;IGKV3D-7**	**Immunoglobulin Kappa Variable**	-1,01	-0,59	-0,69	-0,62	-0,69	-0,48	0,29	-0,39	1,52	0,65	2,02	1
**ITIH1**	**Inter-alpha-trypsin inhibitor heavy chain H1**	-0,67	-0,51	-0,74	-0,45	-0,20	-0,19	-0,13	-0,24	2,79	0,71	-0,36	1
**JCHAIN;IGJ**	**Immunoglobulin J chain**	-1,16	-0,69	-0,74	-0,57	-0,12	-0,63	0,11	0,17	2,01	1,35	0,14	1
**APOH**	**Apolipoprotein H**	-1,41	-0,98	-1,07	-1,08	0,21	0,55	0,18	0,20	1,45	1,36	0,60	2
**IGKC**	**Immunoglobulin Kappa Constant**	-1,38	-0,99	-1,05	-0,98	0,15	0,40	0,44	0,12	1,69	1,31	0,30	2
**IGKV2-40;IGKV2D-28;IGKV A18;IGKV2D-26;IGKV2D-30;IGKV2D-29**	**Immunoglobulin Kappa Variable**	-1,39	-0,92	-1,12	-1,22	0,08	0,47	0,45	0,29	1,45	1,08	0,83	2

**L1TD1**	**LINE-1 type transposase domain-containing protein 1**	-1,35	-1,21	-1,08	-1,22	0,41	0,71	0,62	0,34	1,11	1,20	0,47	2
**TF**	**Serotransferrin**	-1,28	-1,06	-1,03	-1,13	0,14	0,18	0,24	0,15	1,29	1,34	1,15	2
**IGHM**	**Immunoglobulin Heavy Constant Mu**	-1,60	-0,78	-0,97	-0,99	0,19	0,61	0,69	0,31	1,87	0,67	-0,01	2
**LIMS1**	**LIM Zinc Finger Domain Containing 1**	-1,30	-0,97	-1,18	-1,22	0,07	0,40	1,07	0,12	1,32	0,88	0,81	2
**AZGP1**	**Zinc-alpha-2-glycoprotein**	-1,49	-1,74	-1,26	-1,47	0,13	0,94	0,45	0,40	0,77	0,84	0,81	2
**TTR**	**Transthyretin**	-1,20	-0,97	-0,92	-0,81	0,26	0,11	0,60	-0,54	1,15	1,45	0,99	2
**IGHG1**	**Ig gamma-1 chain C region**	-1,35	-0,98	-0,95	-0,79	0,46	-0,56	0,38	-0,02	1,00	1,67	1,12	2
**HNRNPK**	**Heterogeneous nuclear ribonucleoprotein K**	0,81	0,67	0,67	0,52	-1,52	-0,44	-0,61	-1,29	1,33	0,71	0,50	3
**OPTN**	**Optineurin**	0,36	0,87	0,63	0,75	-1,24	-1,11	-1,22	-1,13	1,28	0,98	-0,17	3
**AGT**	**Angiotensinogen**	0,62	0,41	0,63	0,84	-1,25	-0,93	-1,19	-1,30	1,08	1,27	-0,19	3
**APOM**	**Apolipoprotein M**	0,72	0,87	0,63	0,52	-1,42	-1,25	-0,81	-1,43	0,70	1,05	0,41	3
**CLU**	**Clusterin**	0,16	0,60	0,43	0,23	-1,07	-1,20	-1,02	-1,32	1,31	1,44	0,44	3
**APOB**	**Apolipoprotein B**	0,50	0,70	0,65	0,62	-1,38	-1,16	-1,09	-1,30	1,19	0,83	0,45	3
**C4B;C4A**	**Complement C4-B; C4-A**	0,22	0,55	0,53	0,54	-1,17	-0,99	-1,32	-1,36	1,14	1,16	0,70	3
**CHMP4A**	**Charged Multivesicular Body Protein 4A**	0,66	0,37	0,40	0,18	-1,34	-1,57	-1,26	-1,39	1,13	0,50	0,93	3
**APOC1**	**Apolipoprotein C-I**	0,18	0,33	0,57	0,37	-0,80	-0,87	-1,01	-1,84	1,50	0,95	0,64	3
**SERPINA3**	**Serpin Family A Member 3**	0,15	-0,02	0,71	0,08	-1,12	-1,40	-0,77	-1,11	1,50	1,32	0,66	3
**APOA4**	**Apolipoprotein A-IV**	-0,01	0,22	0,19	0,13	-1,11	-0,99	-1,10	-1,20	1,36	1,37	1,15	3
**APOD**	**Apolipoprotein D**	-0,01	0,22	0,20	-0,15	-1,26	-0,84	-0,75	-1,32	1,53	1,43	0,96	3

**PON1**	**Serum paraoxonase/arylesterase 1**	0,07	0,40	0,17	0,25	-1,43	-1,03	-0,68	-1,19	0,65	1,86	0,93	3
**APOL1**	**Apolipoprotein L1**	-0,22	0,61	-0,26	0,12	-1,06	-0,83	-0,34	-1,29	1,25	2,08	-0,07	3
**C4BPA**	**Complement Component 4 Binding Protein Alpha**	-0,92	0,22	0,28	0,67	-0,85	-1,09	-0,52	-1,29	1,71	0,54	1,24	3
**PCYOX1**	**Prenylcysteine oxidase 1**	-0,07	-0,49	-0,07	-0,01	-0,42	-0,35	-0,59	-0,34	0,69	0,72	0,39	3
**APOC3**	**Apolipoprotein C-III**	0,35	0,45	0,52	0,37	0,09	-0,93	-0,37	-0,79	0,69	0,84	0,50	3
**VWF**	**von Willebrand factor**	1,14	1,35	1,20	1,24	-0,71	-0,55	-0,50	-0,45	-0,59	-0,96	-1,17	4
**FCN2**	**Ficolin-2**	0,87	1,61	0,44	1,44	-0,26	-0,13	-0,51	-0,94	-0,30	-0,57	-1,65	4
**SAA2-SAA4;SAA4**	**Serum amyloid A-4 protein**	0,97	1,13	1,15	1,00	-0,35	-0,23	-0,69	-0,25	-0,27	-2,14	-0,33	4
**SNX25**	**Sorting nexin-25**	0,35	1,09	1,58	0,86	-1,15	-0,73	-1,40	-1,23	0,14	0,32	0,17	4
**FNBP4**	**Formin-binding protein 4**	0,92	1,14	1,09	1,01	-1,26	-0,94	-1,14	-1,24	0,17	0,51	-0,24	4
**APOC4-APOC2;APOC2**	**Apolipoprotein C-II;Proapolipoprotein C-II**	1,02	1,39	1,34	1,05	-0,85	-0,75	-0,98	-1,21	-0,23	-0,14	-0,63	4
**APOE**	**Apolipoprotein E**	1,15	1,41	1,27	1,08	-0,88	-0,82	-0,72	-0,97	-0,28	-0,36	-0,88	4
**LBP**	**Lipopolysaccharide-binding protein**	1,04	1,25	1,20	1,20	-0,91	-1,10	-1,03	-1,11	-0,01	-0,17	-0,36	4
**LPA**	**Lipoprotein(A)**	1,28	1,22	1,15	1,12	-0,79	-1,34	-0,89	-0,89	-0,14	-0,30	-0,42	4
**SAA1;SAA2**	**Serum amyloid A-1; A-2 protein**	1,06	1,03	1,02	1,02	-1,14	-1,14	-1,13	-1,14	-0,49	-0,63	-0,72	4
**HELZ**	**Helicase with zinc finger domain**	0,38	0,11	1,83	1,43	-0,99	-1,33	-0,99	-0,66	-0,28	-0,42	-0,07	4
**CD9**	**CD9 antigen**	-0,22	0,04	-0,11	-0,06	0,90	1,10	1,21	1,10	-1,19	-1,40	-1,37	5
**PF4;PF4V1**	**Platelet Factor 4**	-0,19	-0,07	0,06	-0,05	0,98	1,21	1,18	0,86	-1,30	-1,12	-1,57	5

**F11R**	**Junctional adhesion molecule A**	0,15	-0,20	0,14	-0,28	1,02	1,03	1,14	1,00	-1,19	-1,34	-1,49	5
**PARVB**	**Beta-parvin**	0,15	-0,22	-0,19	0,26	0,96	1,09	1,05	0,98	-1,20	-1,24	-1,63	5
**PPBP**	**Platelet basic protein**	-0,42	-0,42	-0,32	-0,37	0,99	1,03	1,25	1,01	-1,30	-1,44	-1,37	5
**ITGA6**	**Integrin alpha-6**	-0,46	-0,23	-0,25	0,26	0,60	1,18	1,37	1,21	-1,08	-1,18	-1,40	5
**SNAP23**	**Synaptosomal-associated protein 23**	0,22	-0,17	-0,19	-0,23	0,67	0,81	0,73	1,39	-1,45	-1,78	-1,61	5
**CD36**	**CD36 antigen**	-0,35	-0,49	-0,54	0,29	1,29	1,13	1,11	1,03	-1,13	-1,20	-1,15	5
**PECAM1**	**Platelet endothelial cell adhesion molecule**	-0,34	0,13	-0,32	-0,21	0,72	0,66	1,31	1,12	-1,34	-1,53	-1,72	5
**YWHAG**	**14-3-3 protein gamma**	-0,21	-0,08	-0,16	-0,11	0,96	0,54	1,17	1,09	-1,48	-1,72	-1,60	5
**ITGB1**	**Integrin beta-1**	-0,05	-0,17	0,17	-0,04	1,15	0,39	1,29	1,20	-1,09	-1,19	-1,66	5
**GNAI2;GNAS; GNAT2; GNAO1;GNAL;GNAT1; GNAI1;GNAT3;GNAI3**	**G Protein Subunit Alpha**	0,11	0,03	-0,03	-0,03	1,04	0,44	1,27	1,20	-0,95	-1,41	-1,66	5
**GNB4;GNB3; GNB2;GNB1**	**G Protein Subunit Beta**	-0,13	0,02	-0,03	-0,07	1,21	0,55	1,61	1,47	-1,01	-1,22	-1,20	5
**RSU1**	**Ras suppressor protein 1**	-0,03	0,14	0,05	0,08	0,81	0,88	0,91	0,70	-1,74	-1,76	-1,79	5
**TPM1**	**Tropomyosin alpha-1 chain**	0,07	0,09	-0,09	-0,04	0,75	0,94	0,98	0,75	-1,64	-1,83	-1,73	5
**GP1BA**	**Glycoprotein Ib Platelet Subunit Alpha**	0,07	0,15	0,15	0,11	0,72	0,76	0,81	0,82	-1,60	-1,99	-1,79	5
**HLA-B;HLA-C;HLA-A**	**HLA class I histocompatibility antigen, B-7 alpha chain**	0,30	0,04	0,44	0,21	1,04	0,09	1,14	1,05	-1,26	-1,47	-1,56	5

**ALDOA**	**Fructose-bisphosphate aldolase A**	0,11	-0,13	0,28	0,31	0,39	0,83	0,96	0,82	-1,86	-1,78	-1,70	5
**GP1BB**	**Platelet glycoprotein Ib beta chain**	0,16	0,30	0,22	0,28	0,80	0,85	0,95	0,91	-1,42	-1,47	-1,58	5
**VCL**	**Vinculin**	0,18	0,26	0,16	0,23	0,82	1,00	0,94	0,84	-1,51	-1,35	-1,57	5
**FERMT3**	**Fermitin family member 3**	0,11	0,23	0,26	0,30	0,89	0,69	0,99	0,98	-1,42	-1,53	-1,50	5
**GP9**	**Platelet glycoprotein IX**	0,16	0,38	0,24	0,14	0,80	0,88	0,93	0,92	-1,41	-1,64	-1,40	5
**PPIA**	**Peptidyl-prolyl isomerase A**	0,21	0,32	0,30	0,29	0,79	0,88	0,90	0,82	-1,41	-1,56	-1,55	5
**ACTB;ACTG1**	**Actin Beta**	0,16	0,31	0,24	0,24	0,79	0,92	0,93	0,88	-1,34	-1,53	-1,59	5
**YWHAZ**	**14-3-3 protein zeta/delta**	0,18	0,37	0,30	0,29	0,79	0,84	0,91	0,81	-1,35	-1,42	-1,73	5
**FLNA**	**Filamin-A**	0,18	0,43	0,26	0,29	0,81	0,84	0,88	0,81	-1,40	-1,33	-1,76	5
**PFN1**	**Profilin-1**	0,26	0,36	0,38	0,35	0,81	0,82	0,86	0,72	-1,37	-1,47	-1,71	5
**TUBA1A;TUBA1B;TUBA1C;TUBA3E**	**Tubulin alpha-1A; 1B; 1C; 3E chain**	0,32	0,45	0,02	0,32	0,81	0,80	0,93	0,85	-1,47	-1,55	-1,47	5
**ITGA2B**	**Integrin alpha-IIb**	0,05	0,15	0,15	0,13	0,92	0,96	1,02	0,94	-1,30	-1,43	-1,58	5
**ITGB3**	**Integrin Subunit Beta 3**	0,10	0,16	0,19	0,13	0,96	0,90	0,99	0,91	-1,45	-1,38	-1,54	5
**RAB1B;RAB1A; RAB15;RAB8B;RAB1C; RAB13;RAB8A**	**Member RAS Oncogene Family**	0,05	0,10	0,13	0,10	0,85	1,02	1,05	0,96	-1,16	-1,48	-1,63	5
**RAP1B;RAP1A**	**Member RAS Oncogene Family**	0,07	0,20	0,15	0,17	0,95	0,85	1,04	0,92	-1,27	-1,46	-1,62	5
**SDPR**	**Serum deprivation-response protein**	0,32	0,03	0,28	-0,09	1,02	1,13	0,45	0,36	-1,62	-1,88	-1,75	5
**CFL1**	**Cofilin-1**	0,26	0,32	0,31	0,29	0,76	0,75	0,83	0,87	-2,06	-0,97	-1,36	5

**F13A1**	**Coagulation factor XIII A chain**	0,22	0,04	0,42	0,42	0,48	1,37	0,88	0,56	-1,36	-1,46	-1,57	5
**FCN3**	**Ficolin-3**	0,46	0,56	0,56	0,52	0,65	0,61	0,67	0,63	-1,51	-1,47	-1,68	5
**B2M**	**Beta-2-microglobulin**	0,42	0,42	0,37	0,36	0,44	0,52	1,15	0,85	-1,44	-1,37	-1,71	5
**TLN1**	**Talin-1**	0,22	0,17	0,25	0,12	0,76	0,81	1,07	0,98	-1,35	-1,35	-1,69	5
**MSN**	**Moesin**	-0,13	-0,04	-0,12	-0,10	0,78	0,63	0,64	1,48	-2,01	-1,13	-1,57	5
**PKM**	**Pyruvate Kinase M1/2**	-0,63	0,03	0,72	0,77	0,52	0,57	1,39	0,68	-1,39	-1,40	-1,26	5
**YWHAH**	**14-3-3 protein eta**	0,26	-0,07	0,31	0,37	0,36	0,39	1,00	0,85	-1,11	-2,34	-1,73	5
**FCER1G**	**High affinity immunoglobulin epsilon receptor subunit gamma**	-0,13	-0,22	-0,30	-0,33	2,23	0,92	0,01	0,87	-0,90	-1,00	-1,17	5
**HSPA7;HSPA6**	**Putative heat shock 70 kDa protein 7**	0,05	0,31	0,15	0,10	1,79	0,50	0,53	0,66	-1,38	-1,61	-1,09	5
**CALM2;CALM1;CALM3; CALML3**	**Calmodulin-like protein 3**	-0,34	0,26	-0,32	0,08	1,73	0,43	0,73	0,49	-1,44	-1,44	-1,63	5
**YWHAE**	**14-3-3 protein epsilon**	-0,14	0,19	-0,22	-0,03	0,23	0,79	0,88	0,73	-2,00	-2,43	-2,22	5
**CORO1A**	**Coronin-1A**	0,02	0,21	0,20	0,02	0,43	0,48	0,64	0,60	-2,80	-2,59	-3,00	5
**CORO1C**	**Coronin-1C**	-0,41	-0,03	0,19	-0,22	0,63	0,99	0,72	0,50	-2,50	-2,37	-2,43	5
**ENO1**	**Enolase**	-0,46	-0,31	-0,48	-0,27	0,77	0,97	0,99	0,83	-2,00	-2,00	-2,05	5
**YWHAQ**	**14-3-3 protein theta**	-0,33	-0,23	-0,38	-0,38	0,40	0,56	1,76	0,53	-1,92	-2,00	-1,80	5
**HSPA8;HSPA2**	**Heat shock cognate 71 kDa protein**	-0,22	-0,07	-0,05	-0,19	0,55	0,93	1,00	1,40	-2,27	-0,37	-0,71	5
**FYB**	**FYN-binding protein 1**	-0,68	-0,68	-0,74	-0,75	0,28	1,30	1,28	1,12	-1,00	-1,00	-1,13	5
**CD47**	**Leukocyte surface antigen CD47**	-0,83	-0,61	-0,65	-0,27	0,71	1,41	0,57	1,17	-1,50	-1,00	-1,25	5

**RGS10**	**Regulator of G-protein signaling 10**	-0,48	-0,31	-0,35	-0,57	0,95	0,95	0,31	0,12	-0,46	-0,75	-1,04	5
**CLIC1**	**Chloride intracellular channel protein 1**	-0,20	-1,64	-0,04	-0,12	1,13	0,50	0,45	1,28	-1,38	-1,38	-1,38	5
**FGB**	**Fibrinogen beta chain**	0,76	0,87	0,86	0,78	0,36	0,32	0,33	0,28	-1,55	-1,55	-1,46	6
**FGG**	**Fibrinogen gamma chain**	0,73	0,81	0,79	0,74	0,36	0,39	0,41	0,37	-1,51	-1,50	-1,59	6
**FGA**	**Fibrinogen alpha chain**	0,74	0,80	0,78	0,75	0,42	0,37	0,35	0,39	-1,45	-1,56	-1,59	6
**C1R**	**Complement C1r**	0,75	0,80	1,09	0,67	0,56	0,24	0,26	-0,02	-1,32	-1,05	-1,97	6
**VCP**	**Valosin Containing Protein**	0,78	0,92	1,03	1,28	0,10	0,20	-0,21	0,03	-1,34	-1,57	-1,22	6
**F5**	**Coagulation factor V**	0,70	1,05	1,03	1,00	0,00	0,21	0,13	0,17	-1,18	-1,43	-1,68	6
**LGALS3BP**	**Galectin-3-binding protein**	0,60	0,82	0,90	0,79	-0,45	-0,43	0,97	0,83	-1,18	-1,28	-1,57	6
**HLA-G**	**Major Histocompatibility Complex, Class I, G**	-1,20	-0,53	-1,08	-0,84	-0,14	0,75	1,63	0,75	1,01	0,47	0,68	7
**DCD**	**Dermcidin**	-1,43	-1,17	-1,49	-1,63	0,17	1,18	1,18	1,17	0,15	0,13	0,60	7
**ZNF585B**	**Zinc Finger Protein 585B**	-0,79	0,12	-0,85	-2,09	-0,31	1,40	1,19	0,70	0,34	0,40	-0,11	7
**HBA1**	**Hemoglobin subunit alpha**	-1,02	-1,02	-1,00	-1,18	0,87	1,06	1,01	1,60	0,05	-0,11	-0,25	7
**HBB;HBD**	**Hemoglobin subunit beta; delta**	-1,00	-0,90	-1,11	-1,18	0,79	1,26	1,10	1,31	0,29	0,03	-0,58	7
**RGS12**	**Regulator of G-protein signaling 12**	-0,94	-0,48	-0,69	-0,84	1,06	1,66	1,02	1,25	0,05	-0,61	-1,27	7
**PRSS3**	**Serine Protease 3**	-1,05	-0,52	-1,03	-1,59	0,99	1,46	0,98	0,01	-0,15	-0,25	-0,05	7
